# DUSP10 Is a Regulator of YAP1 Activity Promoting Cell Proliferation and Colorectal Cancer Progression

**DOI:** 10.3390/cancers11111767

**Published:** 2019-11-09

**Authors:** Marta Jiménez-Martínez, Cristina M. Ostalé, Lennart R. van der Burg, Javier Galán-Martínez, James C. H. Hardwick, Ricardo López-Pérez, Lukas J. A. C. Hawinkels, Konstantinos Stamatakis, Manuel Fresno

**Affiliations:** 1 Department of Cell Biology and Immunology, Centro de Biología Molecular “Severo Ochoa” (CSIC-UAM), 28049 Madrid, Spain; mjimenez@cbm.csic.es (M.J.-M.); jgalan@cbm.csic.es (J.G.-M.); ricardo.lpezprez@gmail.com (R.L.-P.); kstamatakis@cbm.csic.es (K.S.); 2 Department of Molecular Biology, Universidad Autónoma de Madrid (UAM), 28049 Madrid, Spain; 3 Instituto de Investigación Sanitaria de La Princesa (IIS-P), 28006 Madrid, Spain; 4 Department of Development and Regeneration, Centro de Biología Molecular “Severo Ochoa” (CSIC-UAM), 28049 Madrid, Spain; cmostale@cbm.csic.es; 5 Department of Gastroenterology-Hepatology, Leiden University Medical Center, 2333ZA Leiden, The Netherlands; l.r.a.vanderburg@gmail.com (L.R.v.d.B.); J.C.H.Hardwick@lumc.nl (J.C.H.H.); L.J.A.C.Hawinkels@lumc.nl (L.J.A.C.H.)

**Keywords:** protein, cancer, cell contact inhibition, cell proliferation, prognosis

## Abstract

Cell contact inhibition (CCI) is deregulated in cancer. Colorectal cancer (CRC) is the third most commonly diagnosed cancer worldwide. We found that dual-specificity phosphatase 10 (DUSP10) is involved in CRC. DUSP10 overexpression increased the growth of CRC cell lines and mouse xenografts, while the opposite phenotype was observed by DUSP10 silencing. High cell density (HD) induced DUSP10 expression in CRC cell lines, particularly within the nucleus. Yes-associated protein 1 (YAP1) is activated by dephosphorylation, controlling organ growth and CCI, both processes being deregulated in CRC. Expression levels and localization of DUSP10 matched with YAP1 levels in CRC cell lines. DUSP10 and YAP1 co-immunoprecipitated and their interaction was dependent on YAP1 Ser397. The existence of DUSP10 and YAP1 pathway in vivo was confirmed by using a transgenic *Drosophila* model. Finally, in CRC patients’ samples, high levels of nuclear DUSP10 correlated with nuclear YAP1 in epithelial tumor tissue. Strong nuclear DUSP10 staining also correlated with high tumor stage and poor survival. Overall, these findings describe a DUSP10–YAP1 molecular link in CRC cell lines promoting cell growth in HD. We present evidence suggesting a pro-tumorigenic role of nuclear DUSP10 expression in CRC patients.

## 1. Introduction

Colorectal cancer (CRC) is the third most diagnosed cancer worldwide; more than 600,000 people die annually [1]. New treatments have been developed for primary and metastatic CRC, but cure rates and long-term survival have changed little in the past several decades [2]. Statistics indicate that currently available treatment options do not achieve the desired efficiency, requiring the development of new therapeutic strategies in CRC disease [3].

Cell contact inhibition (CCI) regulates cell proliferation and maintains cellular density in tissues. CCI is deregulated in cancer and represents a fundamental distinction between cancerous and normal cells [4]. The Hippo signaling is an evolutionarily conserved pathway implicated in the control of organ size, and its downregulation promotes tumorigenesis [5]. A major downstream effector of this pathway is Yes-associated protein 1 (YAP1), which is regulated by a phosphorylation/dephosphorylation process [6]. YAP1 acts as a co-transcription factor and determines several functions such as organ growth, cell–cell contact sensing, and epithelial architecture [7]. Phosphorylation of YAP1 at Ser127 and Ser397 residues by large tumor suppressor kinase (LATS) is required for its cytoplasmic sequestration and ubiquitin-dependent degradation [8,9]. The activation of YAP1 is essential for CRC initiation, progression, and metastasis [10]. Alteration of YAP1 phosphorylation allows cancerous cells to escape from CCI at high cell density (HD) [9].

The family of dual-specificity phosphatases is a subgroup of phosphatases subclassified by structure, substrate preference, and cellular localization [11]. Among them, there are ten catalytically active mitogen-activated protein kinase (MAPK) phosphatases described in mammals [12]. Dual-specificity phosphatase 10 (DUSP10 or MKP5) is able to dephosphorylate p38 and c-jun N-terminal kinase (JNK). However, it may regulate other targets such as interferon regulatory ractor 3 (IRF3) and extracellular signal-regulated kinase (ERK) by dephosphorylation or association, respectively [13,14]. Recently, DUSP10 has been proposed as regulator of inflammation, immunity, and cancer [15]; although its involvement remains to be explored and confirmed.

Here, we show that DUSP10 has an important role in CRC progression. DUSP10 overexpression and silencing in CRC cell lines regulate in vitro cell proliferation and in vivo xenografts growth in mice. DUSP10 was induced in CRC cell lines in HD. We found that DUSP10 expression level and localization correlated with YAP1. Both proteins co-immunoprecipitated depending on YAP1 Ser397. The functional link of DUSP10–YAP1 was corroborated in a *Drosophila* model with altered Hippo-Salvador-Warts (HSW) pathway activity. Finally, we report an association of nuclear DUSP10 with nuclear YAP1 in CRC patients. Nuclear DUSP10 expression was correlated with high tumor stage and a poor prognosis in a large cohort of CRC patients.

## 2. Results

### 2.1. DUSP10 Regulates Cell Proliferation of CRC Cell Lines In Vitro and In Vivo

To study the role of phosphatase DUSP10 in colon carcinogenesis, we generated CRC cell lines stably overexpressing DUSP10 (Figure S1a) or shRNA-mediated silencing DUSP10 (shDUSP10) (Figure S1c). As a control, we monitored phosphorylated levels of p38 (p-p38). HT29lucD6-DUSP10 decreased p-p38 levels, but not phosphorylated-JNK (p-JNK) (Figure S1b). HT29lucD6-shDUSP10 had the opposite effect on p-p38, while p-JNK did not change (Figure S1d). These results confirmed the efficiency of our cell model in vitro and showed that DUSP10 modulates p38 but not JNK in CRC cells.

**Figure 1:**
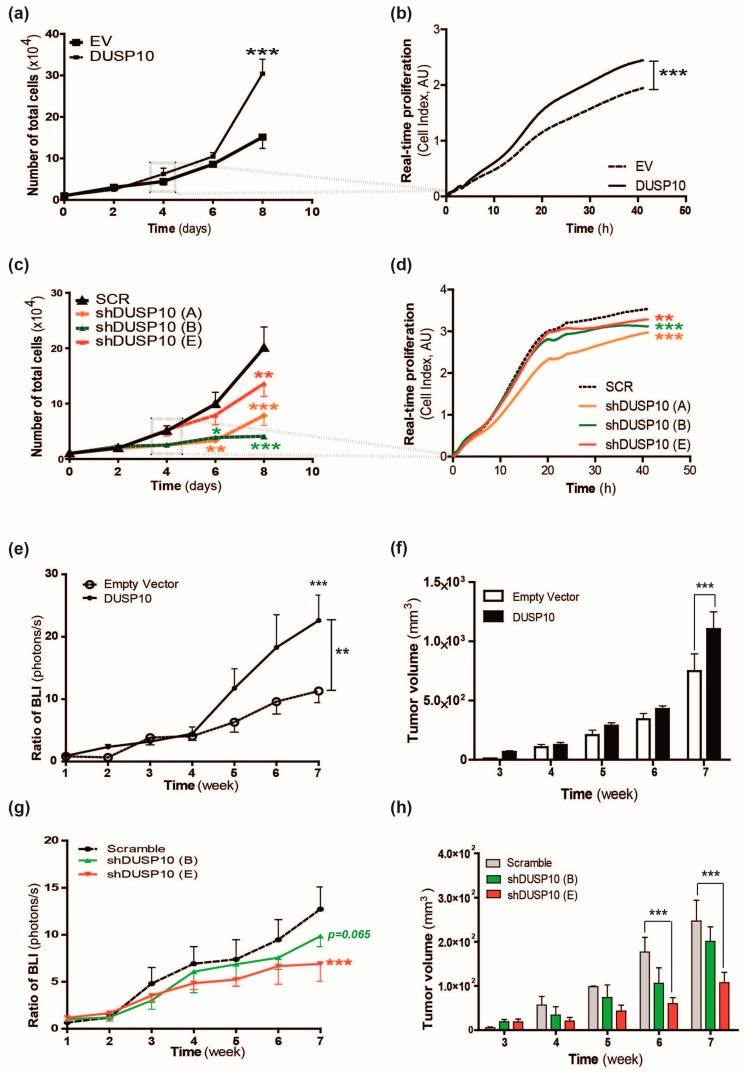
Dual-specificity phosphatase 10 (DUSP10) expression promotes higher colorectal cancer (CRC) cell proliferation and in vivo tumor growth. (**a**) Total cell number of HT29lucD6-DUSP10 was normalized to HT29lucD6-EV. Two-way ANOVA followed by Bonferroni’s post-test (mean ± standard error of mean (SEM); *** *p* < 0.001) and eight independent experiments were performed. (**b**) Growth curves of HT29lucD6-EV and HT29lucD6-DUSP10 for 42 h using real-time proliferation analysis by xCELLigence technology. Linear regression analysis was performed (*** *p* < 0.001). Representative graph of six independent experiments. (**c**) Total cell number of HT29lucD6-shDUSP10 cell lines was normalized to HT29lucD6-SCR. Two-way ANOVA followed by Bonferroni’s post-test (mean ± SEM; * *p* < 0.05, ** *p* < 0.01, *** *p* < 0.001) and seven independent experiments were performed. (**d**) Growth curves of HT29lucD6-shDUSP10 and HT29lucD6-SCR for 42 h using real-time proliferation analysis by xCELLigence technology. Linear regression analysis was performed (** *p* < 0.01, *** *p* < 0.001). Representative graph of three independent experiments. (**e**) Bioluminescence imaging (BLI) of mice xenoinjected with HT29lucD6-DUSP10 and HT29lucD6-EV. Data was normalized to first week post-inoculation for each cell line. Two-way ANOVA followed by Bonferroni’s multiple comparison and linear regression analysis were performed (mean ± SEM; *p* < 0.05; 7–8 mice per group). (**f**) Tumor volume of HT29lucD6-DUSP10 and HT29lucD6-EV xenografts was measured for seven weeks. Two-way ANOVA followed by Bonferroni’s multiple comparison tests were performed (mean ± SEM; *p* < 0.05; five mice per group). (**g**) BLI of mice xenoinjected with HT29lucD6-shDUSP10 and HT29lucD6-SCR. Two-way ANOVA with Bonferroni’s multiple comparison test and linear regression analysis were performed (mean ± SEM; *** *p* < 0.001; eight mice per group). (**h**) Tumor volume of HT29lucD6-shDUSP10 and HT29lucD6-SCR xenografts was measured for seven weeks. Two-way ANOVA and Bonferroni’s multiple comparison test were performed (mean ± SEM; *** *p* < 0.001; four mice per group).

HT29lucD6-DUSP10 displayed a proliferative advantage compared to HT29lucD6-empty vector (EV) as shown by the increased cell number and real-time measurements (Figure 1a,b). These results were reproducible in another CRC cell line, HCT116 overexpressing DUSP10 (HCT116-DUSP10) (Figure S2a,b). The opposite phenotype was observed in silenced DUSP10 cell lines. Although silencing was variable and never complete, all HT29lucD6-shDUSP10 lines had a lower proliferation rate than HT29lucD6-SCR (Figure 1c). The appearance of a plateau phase in sigmoidal growth curves was also delayed in HT29lucD6-shDUSP10 cell lines compared to HT29lucD6-SCR (Figure 1d). Thus, DUSP10 is required for optimal in vitro growth of CRC cell lines.

To investigate the in vivo tumorigenic potential of DUSP10 expression, HT29lucD6 cells were xenografted in athymic nude mice and monitored by bioluminescence imaging (BLI) and volume. The tumor growth of HT29-DUSP10 was higher than the HT29-EV cell line (Figure 1e,f). This effect was also confirmed in the HCT116 cell line (Figure S2c). In contrast, HT29lucD6-shDUSP10 resulted to the opposite effect, with a delayed and reduced tumorigenic capacity in tumor growth (Figure 1g,h). These results supported DUSP10 as a positive cell growth regulator protein in CRC cell lines.

### 2.2. DUSP10 Is Increased in HD and Correlates with YAP1 Expression in CRC Cell Lines

**Figure 2:**
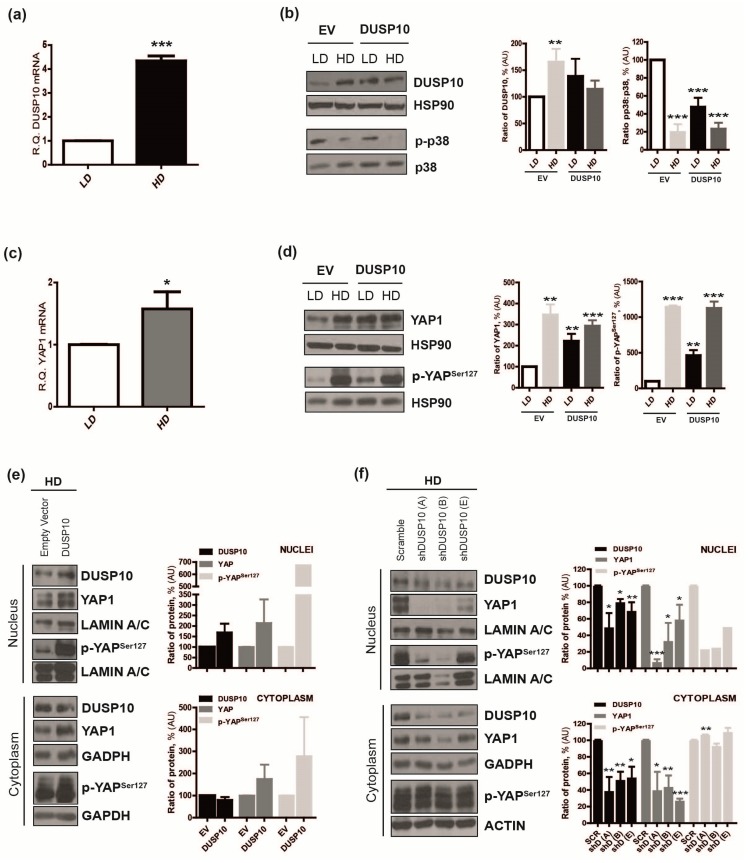
Nuclear DUSP10 and Yes-associated protein 1 (YAP1) are increased in high-density conditions. (**a**) *DUSP10* mRNA was quantified by HT29 in low density (LD) and high density (HD). Student’s *t*-test (mean ± SEM; *** *p* < 0.001) and four independent experiments were performed. (**b**) Expression of DUSP10, p-p38, and p38 of HT29 in LD and HD. (Left) A representative image of five independent experiments. (Right) Quantification of blots performed (mean ± SEM; Student’s *t*-test; ** *p* < 0.01, *** *p* < 0.001). (**c**) *YAP1* mRNA was quantified from HT29 in LD and HD. Student’s *t*-test (mean ± SEM; * *p* < 0.05) and three independent experiments were performed. (**d**) Expression of YAP1 and p-YAP^Ser127^ of HT29 in LD and HD. (Left) A representative image of five independent experiments. (Right) Quantification of blots performed (mean ± SEM; Student’s *t*-test; ** *p* < 0.01, *** *p* < 0.001). (**e**) Expression of DUSP10, YAP1, and p-YAP^Ser127^ proteins from nuclear and cytoplasmic extracts from HT29lucD6-DUSP10 and HT29lucD6-EV in HD. LAMIN A/C and GAPDH (glyceraldehyde 3-phosphate dehydrogenase) are nuclear and cytoplasm control proteins, respectively. (Left) A representative image of three independent experiments. (Right) Quantification of all blots performed (mean ± SEM; Student’s *t*-test; *p* < 0.05). (**f**) Expression of DUSP10, YAP1, and p-YAP^Ser127^ proteins from nuclear and cytoplasmic extracts of HT29lucD6-shDUSP10 and HT29lucD6-SCR in HD. LAMIN A/C and GAPDH/ACTIN are nuclear and cytoplasm control proteins, respectively. (Left) A representative image of three independent experiments. (Right) Quantification of all blots performed (mean ± SEM; Student’s *t*-test; * *p* < 0.05, ** *p* < 0.01, *** *p* < 0.001). Completed immunoblots of Figure 2b,d,e,f are in Figures S8 and S9, respectively.

Growth-modulating effects caused by DUSP10 were more evident in the stationary phase of CRC cell line cultures. Thus, we analyzed DUSP10 expression in response to cell density condition. An induction of *DUSP10* mRNA and DUSP10 protein was observed in HT29 cell line in HD compared to low cell density (LD), matching with a reduction of p-p38 levels (Figure 2a,b). This was also observed in the HCT116 CRC cell line (Figure S3a,b).

The Hippo pathway regulates CCI through YAP phosphorylation [9]. Therefore, we hypothesized that DUSP10 could be a regulator of YAP1 in HD. We observed an increase in the mRNA levels of *YAP1* in HD compared to LD in both HT29 and HCT116 cell lines (Figure 2c, Figure S3c). A huge increase of YAP1 protein and YAP phosphorylation at Serine 127 (p-YAP^Ser127^) in HD over LD was detected. We also observed that DUSP10 overexpressing CRC cell lines, HT29-DUSP10 and HCT116-DUSP10, showed higher levels of YAP1 and p-YAP^Ser127^ than EV cells in LD, that were further augmented by HD (Figure 2d, Figure S3d).

Next, nuclear and cytoplasmic distribution of DUSP10 and YAP1 was analyzed in HD. We observed that nuclear YAP1 was increased in HT29lucD6-DUSP10 compared to EV cells. This increase was also detected in the cytoplasm. We detected the YAP1 localization in CRC cell lines by immunofluorescence (IF) (Figure S4a,b), but the quality was not good enough to have clear conclusions. We also analyzed p-YAP^Ser127^ by western blot (WB). As expected, p-YAP^Ser127^ was mostly detected in the cytoplasm of all cell lines. However, DUSP10 overexpression caused a striking increase in p-YAP^Ser127^ in the nucleus. (Figure 2e). Concomitantly, HT29lucD6-shDUSP10 resulted in the opposite phenotype, displaying a reduction of nuclear YAP1 and also in the cytoplasm. p-YAP^Ser127^ levels were reduced in silenced DUSP10 cell lines in the nucleus. However, DUSP10 knockdown neither affected the amount nor the smearing of p-YAP^Ser127^ in the cytoplasm (Figure 2f). The above results indicate that DUSP10 levels correlated with YAP1 levels, particularly matching in HD.

**Figure 3:**
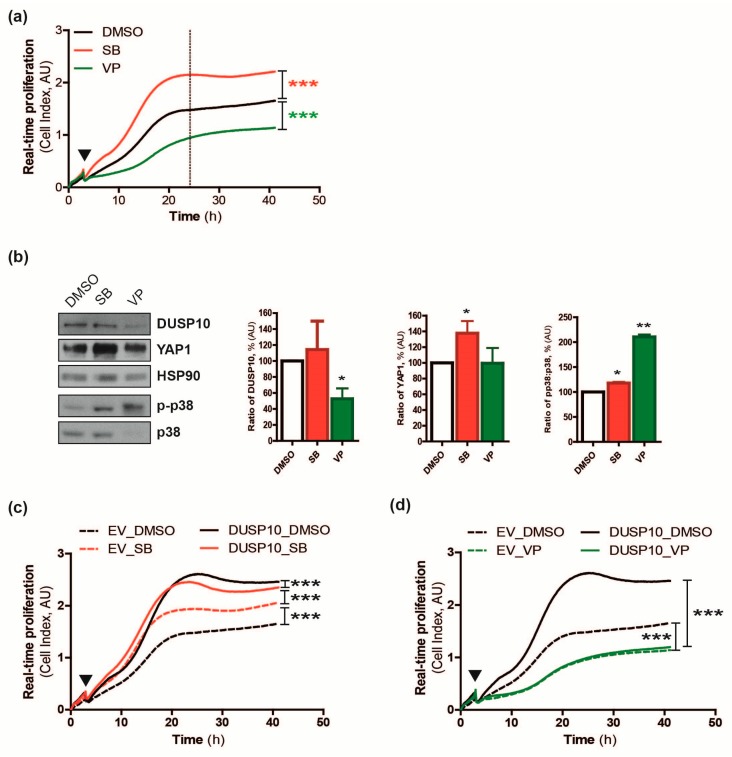
A YAP inhibitor prevents DUSP10-enhanced proliferation. (**a**) Growth curves of HT29 proliferative response to SB239063 (SB 1 µM) and verteporfin (VP 1 µM) for 40 h after 2 h seeding (▼) using real-time proliferation analysis by xCELLigence technology. Linear regression analysis was performed (*** *p* < 0.001). Representative graph of four independent experiments. (**b**) Expression of DUSP10, YAP1, p-p38, and p38 protein levels in HT29 treated with SB (1 µM) and VP (1 µM) for 24 h (dotted line within Figure 3a). (Left) A representative image of three independent experiments. (Right) Quantification of all blots performed (mean ± SEM; Student’s *t*-test; * *p* < 0.05, ** *p* < 0.01). (**c**) Growth curves of HT29-EV and HT29-DUSP10 proliferative response to SB 1 µM and DMSO for 40 h after 2 h seeding (▼) using real-time proliferation analysis by xCELLigence technology. Linear regression analysis was performed (*** *p* < 0.001). Representative graph of three independent experiments. Completed immunoblots are in Figure S10. (**d**) Growth curves of HT29-EV and HT29-DUSP10 proliferative response treated with VP 1 µM and DMSO for 40 h after 2 h seeding (▼) using real-time proliferation analysis by xCELLigence technology. Linear regression analysis was performed (*** *p* < 0.001). Representative graph of three independent experiments.

Our results confirmed DUSP10 activity on p38 and pointed to a possible regulation of YAP1 in CRC cell lines. For this reason, we studied a possible relationship between all of them in HD using a selective p38α/β inhibitor (SB239063, SB) and a blocker of YAP1 activity (verteporfin, VP) [16,17]. We observed higher proliferation immediately after SB addition, while the growth was drastically reduced after VP treated HT29 and HCT116 cells (Figure 3a, Figure S5a). An increase of DUSP10 and YAP1 protein levels, and to a lesser extent in p-p38/p38 ratio occurred in growing HT29 and HCT116 cells upon SB treatment. In contrast, VP treatment reduced DUSP10 and p38 protein levels and strongly increased p-p38/p38 ratio (Figure 3b, Figure S5b). The enhancing effect of p38 inhibitor on cell proliferation was not observed in HT29-DUSP10 cells, which had a higher proliferative rate than HT29-EV plus SB, even in the late phase when cells reached HD (Figure 3c). Remarkably, the VP inhibitory effect and YAP1 siRNA action were not reversed by DUSP10 overexpression (Figure 3d, Figure S5c), suggesting that DUSP10 growth effect requires YAP1. Thus, pharmacological inhibitors confirmed a molecular link between DUSP10, YAP1, and p38 regulating cell proliferation of CRC cell lines.

### 2.3. DUSP10 Interacts with YAP1 through Ser397 Residue

**Figure 4:**
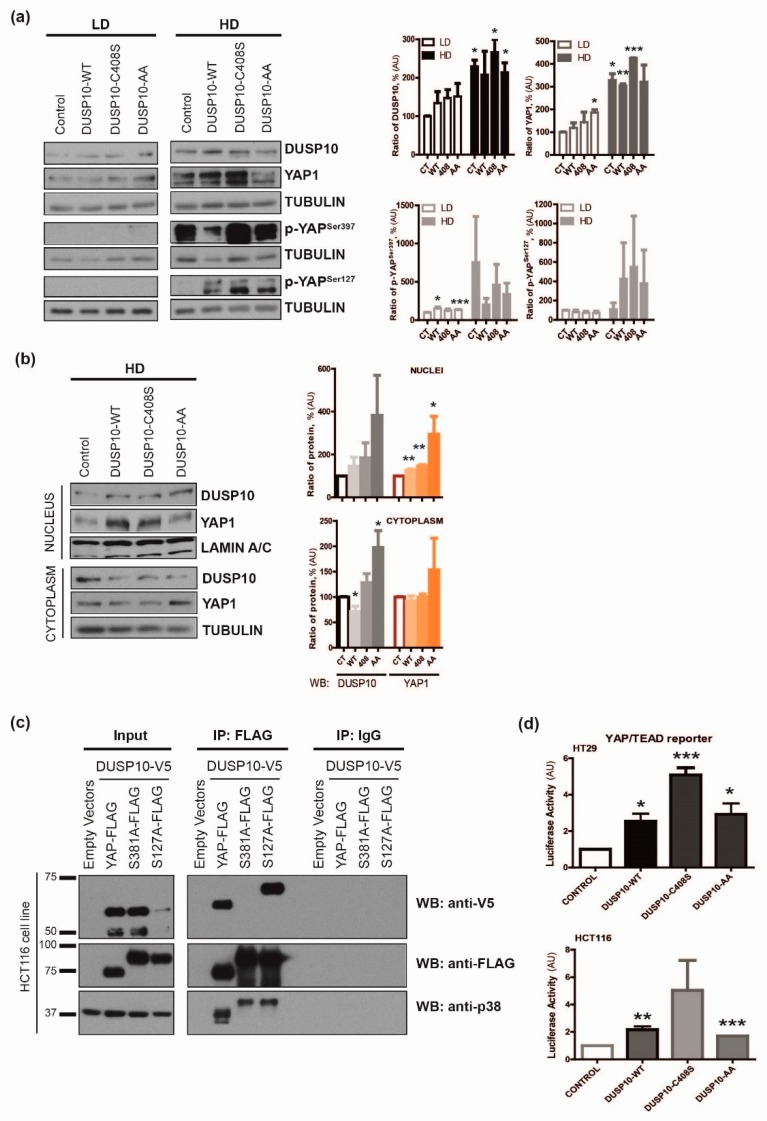
DUSP10 interacts with YAP1 through Ser397 residue. (**a**) Expression of DUSP10, YAP1, p-YAP^Ser397^, and p-YAP^Ser127^ proteins in DUSP10-wild type (DUSP10-WT), phosphatase catalytic site mutant (DUSP10-C408S), and p38 binding site mutant (DUSP10-AA) HT29 cell line in LD and HD. TUBULIN is the control protein. (Top) A representative image of three independent experiments. (Bottom) Quantification of all blots performed (mean ± SEM; Student’s *t*-test; * *p* < 0.05, ** *p* < 0.01, *** *p* < 0.001). (**b**) Analysis of DUSP10, YAP1, and p-YAP^Ser127^ proteins from nuclei and cytoplasm extracts of HT29 DUSP10 mutant cell lines in HD. LAMIN A/C and TUBULIN are used as nuclear and cytoplasm control proteins, respectively. (Left) A representative image of three independent experiments. (Right) Quantification of all blots performed (mean ± SEM; Student’s *t*-test; * *p* < 0.05, ** *p* < 0.01). (**c**) Immunoprecipitation of YAP-FLAG and detection of DUSP10 and p38 in HCT116. DUSP10-V5 and YAP-FLAG plasmids were co-transfected into the cell line and detected by anti-FLAG and anti-V5 antibodies, respectively. YAP1 wild type (YAP1-FLAG) and mutant (S381A-FLAG, S127A-FLAG) plasmids were immunoprecipitated with anti-FLAG. Representative images of three independent experiments. (**d**) Relative luciferase activity of the 8xGTII-luc (YAP/TEAD binding element reporter) was measured in HD, responding to DUSP10 overexpression and DUSP10 mutant constructs. HT29 (Top graph) and HCT116 (Bottom graph) were transiently transfected with the indicated plasmids and its control constructs. Student’s *t*-test (mean ± SEM; ** *p* < 0.01, *** *p* < 0.001) and three independent experiments were performed. Completed immunoblots of Figure 4a–c are in Figures S11 and S12, respectively.

To further address the DUSP10 role on YAP1 activity, we generated HT29lucD6 cell lines overexpressing DUSP10 mutants, without p38 binding capacity (DUSP10-AA) or without phosphatase catalytic activity (DUSP10-C408S). An increase of total YAP1 protein levels was observed with both mutants and with DUSP10 wild type (DUSP10-WT) in LD. EV cells had high levels of p-YAP^Ser397^ in HD, as expected. This phosphorylation was reduced in DUSP10-WT and DUSP10-AA cells, but remarkably not reduced in phosphatase inactive mutant cells. In contrast, all DUSP10 overexpressing cell lines had a similar increase in p-YAP^Ser127^ (Figure 4a). Upon HD, all DUSP10 overexpressing cell lines had higher YAP1 protein levels in the nucleus compared to control cells (Figure 4b).

We hypothesized whether DUSP10 and YAP1 could co-immunoprecipitate. We used a S381A mutant of YAP1 isoform 2 (equivalent of Ser397 of YAP1 isoform1) and S127A mutant of YAP1 proteins [18]. We co-transfected DUSP10 (DUSP10-WT-V5) with YAP1 (YAP1-FLAG) or with S381A YAP mutant (S381A-FLAG) or with S127A YAP mutant (S127A-FLAG) plasmids in HCT116 (Figure S6a,b). DUSP10-V5 co-immunoprecipitated with YAP1 and S127A-YAP but not with the S381A-YAP mutant. We also observed all YAP constructs co-immunoprecipitated with p38 (Figure 4c). Moreover, we studied DUSP10 effect on YAP1 transcriptional activity by using a YAP/TEAD (transcriptional enhanced associate domain) reporter plasmid (Figure S6c). Transient transfection of DUSP10-WT or any DUSP10 mutant resulted in a higher YAP/TEAD transcriptional activity in both HT29 and HCT116 cells compared to control plasmids (Figure 4d). Thus, DUSP10 was able to interact with YAP1 through Ser381 (Ser397 in YAP1 isoform1) and promote YAP/TEAD-dependent gene expression in CRC cell lines.

### 2.4. DUSP10 Regulates the Hippo Pathway in Drosophila

**Figure 5:**
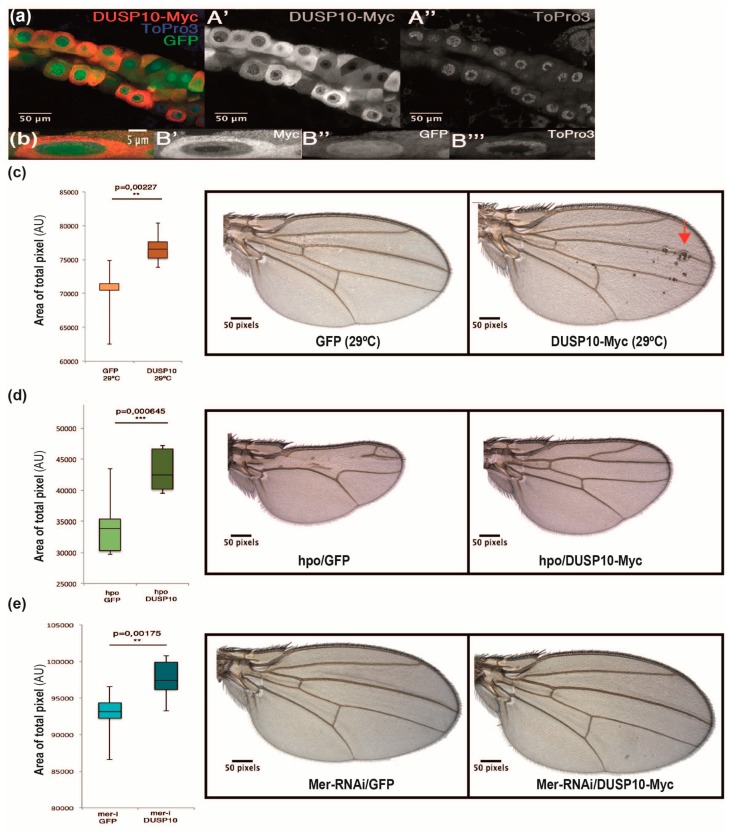
Effect of DUSP10 expression on Hippo-Salvador-Warts pathway in *Drosophila melanogaster*. (**a**) Subcellular localization of DUSP10-Myc (red) in salivary gland cells in *sal^EPv^-Gal4 UAS-GFP/+; UAS-DUSP10-Myc/+* flies. ToPro3 (blue) was used as DNA marker and nuclei were detected by GFP (green) expression. Bars, 50 µm. (**b**) Higher magnification of a salivary gland cell from *sal^EPv^-Gal4 UAS-GFP/+;UAS-DUSP10-Myc/+* individuals. Transversal section was represented. Bars, 5 µm. (**c**) Quantification of wing surfaces from anterior margin to L5 vein in wild type *sal^EPv^-Gal4 UAS-GFP/+* (GFP) and *sal^EPv^-Gal4/+;UAS-DUSP10-Myc/+* (DUSP10-Myc) individuals. Representative images of wing surfaces of both genotypes. Some cell clusters were detected between the two epithelial wings’ surfaces (red arrow). (**d**) Quantification of wing surfaces from margin to L5 vein in *Ste*-20 protein kinase *hippo* overexpressing alone (*sal^EPv^-Gal4 UAS-GFP/UAS-hpo,* hpo/GFP) and combined to human DUSP10-Myc (*sal^EPv^-Gal4/UAS-hpo;UAS-DUSP10-Myc/+,* hpo/DUSP10-Myc) individuals. Representative images of wing surfaces for both genotypes. (**e**) Quantification of wing surfaces from margin to L5 vein in Merlin/Nf2 knockdown alone (*sal^EPv^-Gal4 UAS-GFP/+;UAS-Mer-RNAi,* Mer-RNAi/GFP) and combined to human DUSP10-Myc (*sal^EPv^-Gal4/UAS-Mer-RNAi;UAS-DUSP10-Myc/+,* Mer-RNAi;DUSP10-Myc) individuals. Representative images of wing surfaces for both genotypes. All graphs were represented by the media ± standard deviation (SD) (*n* = 10). Student’s *t*-test was performed to compare genotypes (** *p* < 0.01; *** *p* < 0.001).

In order to confirm DUSP10 and YAP1 functional relationship, we used a *Drosophila* in vivo model. The *Drosophila* genome contains several DUSP proteins, but none of which corresponds to an orthologue of DUSP10 [19]. We expressed the human DUSP10 sequence fused to Myc epitope (DUSP10-Myc) in wing imaginal disc and salivary glands of *nub-Gal4/+;UAS-DUSP10-Myc/+* and *sal^EPv^-Gal4 UAS-GFP/+;UAS-DUSP10-Myc/+* individuals, respectively. We observed that DUSP10-Myc co-localized in the nuclear periphery of salivary gland cells (Figure 5a,b) and imaginal discs (images not shown). Interestingly, we observed that wing size was increased in DUSP10-Myc compared to wild type *sal^EPv^-Gal4 UAS-GFP/+* (green fluorescent protein, GFP) individuals. Concomitantly, we observed small cellular groups in DUSP10-Myc wings, which were dislocated from the epithelium and were localized between the two wing surfaces (Figure 5c). These results suggested that DUSP10 expression caused defects in size and integrity of the imaginal epithelium.

We wondered whether the HSW pathway effect on YAP levels (*Yorkie* protein in *Drosophila*) could be modulated by expressing DUSP10 in vivo [20]. We studied the consequences of DUSP10 expression on sensitized genetic backgrounds by altering the activity of the HSW pathway. The overexpression of *Ste-20* protein kinase *hippo* (*sal^EPv^-Gal4 UAS-GFP/UAS-hpo,* hpo/GFP), a *Drosophila* MST1/2 orthologue, in the central region of the wing imaginal disc resulted in smaller than control (GFP) wings and a defective vein pattern (Figure 5d compared to 5c, left images). When DUSP10-Myc was expressed in a *hippo* overexpression background *sal^EPv^-Gal4/UAS-hpo;UAS-DUSP10-Myc/+* (hpo/DUSP10-Myc), we observed a significant rescue of hpo/GFP phenotype (Figure 5d). We also studied DUSP10-Myc effect on a genetic background with reduced *hippo* activity caused by the loss of Merlin/Nf2 tumor suppressor gene (*sal^EPv^-Gal4 UAS-GFP/UAS-Mer-RNAi,* Mer-RNAi-GFP). Mer-RNAi-GFP wings increased 4.4% of adult wing size compared to GFP wings (Figure 5e compared to 5c, left images). When Mer-RNAi construct was combined with DUSP10-Myc (*sal^EPv^-Gal4/UAS-Mer-RNAi;UAS-DUSP10-Myc/+,* Mer-RNAi/DUSP10-Myc), we observed a significant increase in wing size compared to Mer-RNAi individuals (Figure 5e). In addition, the wing size of Mer-RNAi; DUSP10-Myc individuals showed an increase of 9.2% compared to GFP phenotype (Figure 5e right image compared to 5c left image). These observations suggested that human DUSP10 is able to regulate the activity of *Drosophila* HSW signaling when the normal operation of this pathway was compromised.

### 2.5. DUSP10 Is Increased in CRC Patient Samples

**Figure 6:**
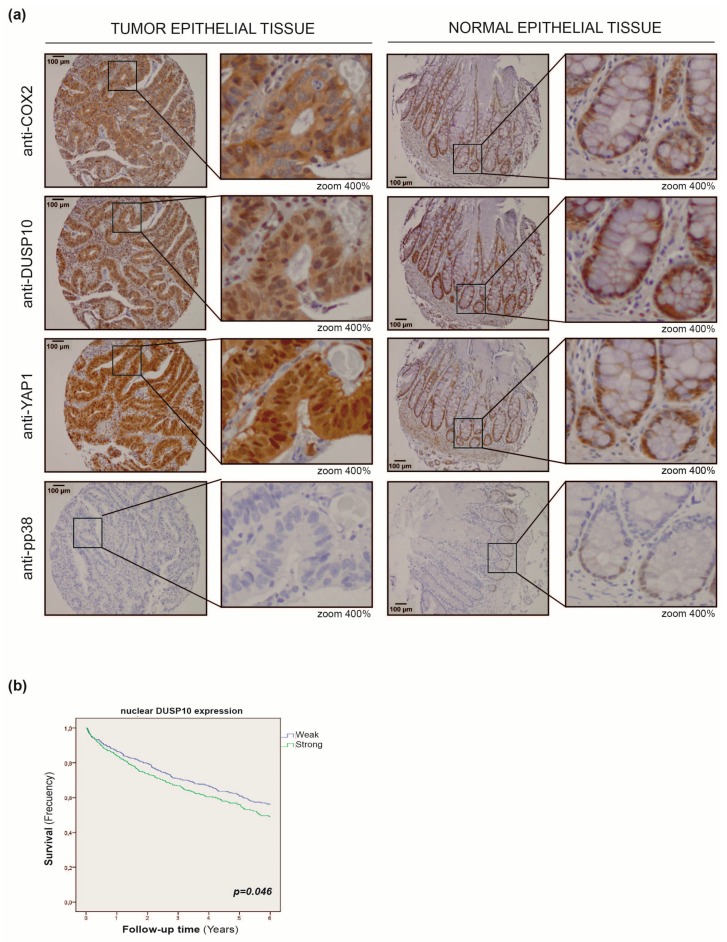
DUSP10 is expressed in colon cancer patients and its nuclear expression is associated with a poor prognosis. (**a**) Representative images of DUSP10, p-p38, and YAP1 staining on a tissue microarray (TMA) in normal epithelial and tumor colon tissue from 73 human patients by immunohistochemistry (IHC). (Left) Image taken at 10× magnification (Bars, 100 μm) and (Right) magnified 400% by zoom. (**b**) Kaplan–Meier survival curve (*p* < 0.05) of 999 colon cancer patients with weak (blue line) and strong (green line) nuclear DUSP10 expression in tumor epithelial tissue for six years.

We addressed the role of DUSP10 in human CRC tumors, by performing immunohistochemistry (IHC) staining on a tissue microarray (TMA) of 73 CRC patients. We found DUSP10 and YAP1 expression higher in tumor samples than normal tissue, but not of p-p38 in most tumor samples (Figure 6a). Moreover, a significant positive correlation between nuclear YAP1 and DUSP10 was found in 63.5% of patients (Table 1).

These IHC results were further confirmed by an in silico gene expression analysis of tumor and normal patient samples performed on the UCSC Xena platform using public access data from The Cancer Genome Atlas colon cancer cohort. An analysis of 430 patient samples showed a significant increase of *DUSP10* mRNA levels in primary CRC compared to normal tissue (Figure S7a). We also found a significant positive correlation of *DUSP10* with *CYR61,* a YAP1-target gene, and mRNA levels (Figure S7b). This in silico analysis suggested a relationship between *DUSP10* and *YAP1* in CRC patients.

### 2.6. Nuclear DUSP10 Expression Correlates with Bad Prognosis in CRC Patients

To corroborate the potential relationship between DUSP10 expression and prognosis, a large cohort of 999 CRC patients was analyzed. Follow up data until six years post-surgery were available. The relationship between cytoplasmic and nuclear DUSP10 expression by IHC and different clinic-pathological parameters was analyzed. We observed an association between DUSP10 expression and tumor stage (Table 2). Specifically, strong nuclear DUSP10 staining was significantly associated with a higher tumor stage compared to weak nuclear DUSP10 staining, adjusted for age, gender, and chemotherapy. At the same time, Cox regression univariate analysis showed a significantly higher hazard ratio (HR) according to strong nuclear DUSP10 expression. In multivariate regression analysis including confounders as age, gender, and chemotherapy, DUSP10 expression maintained a significant HR. Interestingly, no significant relation was found when tumor stage was included as a confounder in the multivariate Cox regression analysis (Table 3). The Kaplan–Meier survival analysis showed a significant decrease of survival in CRC patients with high nuclear DUSP10 expression (Figure 6b). These results indicate that nuclear DUSP10 expression was associated with a higher tumor stage and worse survival of CRC patients.

To further support our results, we performed an in silico analysis using the Oncomine™ database in human CRC samples from Kaiser Colon Collection (GSE5206). A survival analysis of 167 colon cancer patients’ metastasis (GSE17538) showed significantly higher survival in low *DUSP10* expression patients (Figure S7c). Taken together, these data suggest that DUSP10 expression, mostly nuclear, was enhanced in CRC compared to normal tissue and correlated with poor survival.

## 3. Discussion

CCI depends mostly on density-dependent constraints, rather than strict cell–cell contact, with YAP1 signaling as a master regulator of epithelial growth control [21,22]. In LD, YAP1 is activated by dephosphorylation and translocated into the nucleus, acting as a co-transcription factor and activating proliferative genes, while YAP1 is inactivated in HD [9]. In non-transformed epithelial cells, the Hippo pathway is able to respond to HD. However, this equilibrium is severely disturbed in tumor cells.

Here, we report that DUSP10 is a new regulator of YAP1 in CRC. DUSP10 overexpression promoted higher growth of CRC cell lines, both in vivo and in vitro, whereas silencing DUSP10 decreased these effects. Interestingly, a minimal cell confluence was necessary to reveal the cell growth effect of DUSP10 expression, suggesting that CCI may be altered by DUSP10 expression in CRC cell lines. Both DUSP10 and YAP1 were found to accumulate in the nucleus of CRC lines in HD (in contrast with non-transformed cells where YAP1 is degraded). Importantly, variation of DUSP10 levels directly affected YAP1 expression levels, being more evident within the nucleus. This suggests that nuclear YAP1 localization in HD is strongly dependent on DUSP10 expression in CRC cell lines.

The phosphorylation of YAP1 of Ser127 and Ser397 is required for its cytoplasmic sequestration and ubiquitin-dependent degradation [8,9,23]. We found that cytoplasmic p-YAP^Ser127^ levels were increased in CRC lines in HD. We also observed an increase of nuclear p-YAP^Ser127^ levels in DUSP10 overexpressing cells in HD indicative that “canonical YAP inactivation pathway” is disturbed by DUSP10 expression. Interestingly, our results showed that increased p-YAP^Ser397^ levels in HD were drastically reduced by DUSP10 overexpression. DUSP10 mutants suggest that DUSP10 phosphatase activity may mediate a direct p-YAP^Ser397^ dephosphorylation in HD, whereas nuclear YAP1 or p-YAP^Ser127^ accumulation is mostly independent of DUSP10 phosphatase activity or its p38 interaction site. Furthermore, we found that DUSP10 physically interacts with YAP1, which seems to be dependent on p-YAP^Ser397^.

We observed that p38 was also co-immunoprecipitated with YAP1, indicating the possibility of a multi-complex. Some authors have proposed that YAP1 dysregulation promotes tumor development through a p38 regulation of cAMP response element-binning (CREB) transcription factor [24]. Thus, a possibility could be that DUSP10 effect on YAP1 activity may be associated to p38 dephosphorylation [25]. However, the fact that all DUSP10 constructs (mutated or not) activate YAP/TEAD-dependent transcription suggests a model in which DUSP10 independently of p38 interaction site or phosphatase activity may be required for nuclear YAP1 accumulation (even in the form of p-YAP^Ser127^), acting as a scaffold protein for YAP1. In addition, DUSP10 phosphatase would be required for Ser397 YAP dephosphorylation. In this scenario, both activities may cooperate to avoid YAP1 cytoplasmic degradation and promoting YAP1 nuclear accumulation/stabilization and transcriptional activity.

A functional interaction of YAP1 with DUSP10 was further confirmed in a heterologous model, where human DUSP10 expression was able to regulate the activity of *Drosophila* Hippo/Yorkie signaling. This is a well-conserved growth control pathway from *Drosophila* to mammals. Both the mutation in *Drosophila* homolog of YAP (*Yki*) or an excessive *Yki* phosphorylation, lead to its cytoplasmic retention and reduced organ size [6]. p38 activity is not required for wing formation in *Drosophila*, since the overexpression of D-p38b dominant negative molecules (p38β homologue in mammals) induce ectopic formation and size changes of veins in the wing [26]. Our results points to a direct DUSP10 action on the wing cell growth controlling Hippo pathway, which was p38-independent, which is in line with the evidence obtained in CRC lines.

Our cohort of CRC patients showed a strong DUSP10 protein expression in CRC, which was co-expressed with YAP1 in epithelial tumor tissue. Regarding a possible interaction of DUSP10-YAP1, Konsavage et al. reported that 86% of CRC tumors showed nuclear localization of YAP1 [27]. A functional association of high nuclear DUSP10 levels with nuclear YAP1 expression was also observed in tumor cells of CRC patient samples. This is also in agreement with the reported correlation of high nuclear YAP1 expression with poor patient outcome and tumor stage of CRC [10,28,29,30]. Regarding p38, other authors have described that lower p38 expression in colon polyps epithelial tumor cells and p38 inactivation in crypt enterocytes are related to reduced tumor formation [31,32]. However, analysis of our patient cohort showed no correlation of DUSP10 expression with p38 dephosphorylation in epithelial tumor tissue, further supporting another mechanism to explain the DUSP10 tumor promoting effect.

## 4. Materials and Methods

### 4.1. Cell Lines and Cell Culture

HT29 (human epithelial colorectal adenocarcinoma) cell line was obtained from the ATCC^®^ (LGC Standards, Barcelona, Spain). HCT116 (human epithelia colorectal carcinoma) cell line was obtained from Centro de Investigaciones Biológicas (Madrid, Spain). SW480 and SW620 cell lines [33] were obtained from Instituto de Investigaciones Biomédicas (Madrid, Spain). HEK-293T (human epithelia embryonic kidney) cell line was obtained from Centro de Biología Molecular ‘Severo Ochoa’ (Madrid, Spain) and used to generate lentiviral particles. HT29lucD6 cell line (Xenogen Corporation, Hopkinton, MA, USA) constitutively expresses the luciferase reporter, whose expression was selected with 200 µg/mL G418 (Invitrogen, Carlsbad, CA, USA).

Cell lines were grown in MEM 5% fetal bovine serum (FBS) (Gibco, Waltham, MA, USA) (HT29 and HCT116) and DMEM 10% FBS (HEK-293T) supplemented with non-essential amino acids, 0.01% sodium pyruvate, 2 mM glutamine, 100 U/mL penicillin, and 100 µg/mL streptomycin at 37 °C in a 5% CO_2_ humidified atmosphere. Mycoplasma contamination was tested periodically.

### 4.2. Transient Expression Constructs

FLAG or V5 tag plasmids and Universal Negative Control or YAP siRNA (Appendix A) were (co-) transfected by Lipofectamine^®^ 2000 Transfection Reagent (Invitrogen), according to the manufacturer’s instructions.

### 4.3. Stable Expression Constructs

DUSP10 overexpressing and DUSP10 knockdown cell lines were generated by lentiviral particle transduction carrying construct genes or empty vector/scramble basically as described elsewhere [34]. All of the following plasmids are described in Appendix A. Human *DUSP10* cDNA (SC31766, OriGene Technologies, Rockville, MD, USA) was subcloned in the pLenti-CMV/TO-Hygro vector using specific primers. pLenti-KO.1-Puro carrying specific shRNA sequences were used to knockdown DUSP10 constructs (shDUSP10). DUSP10 overexpressing and shDUSP10 cells were selected using 200 µg/mL hygromycin B (Invitrogen) and 4 µg/mL puromycin (Invitrogen), respectively. DUSP10 overexpressing mutants was generated in pLX304-V5 vector with specific DUSP10 guides (Appendix B) using Gateway™ Cloning Technology (Invitrogen), according to the manufacturer’s instructions. These cells were selected by 4 µg/mL blasticidin (Invitrogen). The mutation in the phosphatase catalytic site and p38 interaction site of DUSP10 were performed by Cys 408 codon mutation and Arg 203/204 mutation using specific guides, respectively, as described in Appendix B.

### 4.4. Cell Proliferation Assays

Cells were seeded in 6-well plates at a density of 1 × 10^4^ cells/well. During the following eight days, cells were trypsinized and counted every two days using the hemocytometer chamber.

Real-time proliferation assays were measured by cell analyzer xCELLigence technology (Roche and ACEA Biosciences, San Diego, CA, USA) according to the manufacturer’s instructions. Then, 5 × 10^4^ cells were plated in E-plate 16 wells (Roche, Basel, Switzerland). The growth slope was calculated by measuring cell impedance every 5–15 min, analyzed by Real-Time Cellular Analyzer Software v1.1. (Roche) and converted to a cell index (CI), according to the manufacturer’s instructions. CI was a non-dimensional parameter and represented cell status (number, viability, motility, and adhesion grade).

### 4.5. Cell Density Assay

The cell population from the high-density (HD) condition was obtained 48 h after seeding cells 10 times more concentrated than at low-density (LD). LD cell culture was composed by small colonies corresponding to growing cells (56% G0/G1, 36% S, and 8% G2/M). HD cell culture made a monolayer of G0/G1 arrested cells (72% G0/G1, 19% S, and 9% G2/M).

### 4.6. Luciferase Reporter Activity Assay

Cells were grown in 24-well plates and transfected with DUSP10 mutant plasmids or control vectors, 8xGTII/Luc (YAP-TEAD) construct, and SV40-Renilla control plasmid (used to transfection normalization), using a 9:1:0.1 ratio. Cells were lysed with Passive Lysis Buffer of Dual-Luciferase Reporter Assay Kit (Promega) 48 h later, according to the manufacturer’s instructions. Luciferase activities were measured in a 96-well Nunclon plate in a FLUOstar OPTIMA plate reader (BMG LABTECH, Ortenberg, Germany). Relative luciferase activity was obtained using a ratio of luciferase/renilla and samples/control.

### 4.7. Protein Extraction and Immunoblot Analysis

Cells were lysed with RIPA (radioimmunoprecipitation assay) cell lysis buffer, PhoStop™ phosphatase inhibitor cocktail (Roche) and cOmplet EDTA-free Protease Inhibitor Cocktail (Roche), according to the manufacturer’s instructions. Cell lysates were centrifuged at 12,000× g at 4 °C for 10 min. Protein concentration was determined using Pierce^®^ BCA Protein Assay Kit (Pierce Biotechnology, Waltham, MA, USA) or Bradford assay (BioRad, Hercules, CA, USA), according to the manufacturer’s instructions. Protein was resolved on 10%–12% SDS-PAGE gel and transferred to nitrocellulose membrane (BioRad). Membranes were blocked in 5% BSA (bovine serum albumin)-TBS (tris-buffered saline) with 0.1% Tween20 (TBST) and incubated with a primary specific antibody overnight at 4 °C. The membrane was incubated with horseradish peroxidase-conjugated secondary antibodies for 1 hour at room temperature (RT). Luminescence signal was detected by SuperSignal West Dura Substrate (Pierce Biotechnology) using X-ray films (AGFA, Mortsel, Belgium) and quantified by ImageJ software (National Institutes of Health, NIH). Antibodies are described in Appendix C.

Immunoblots were quantified by ImageJ software. Bands were selected using the square tool. Intensity of bands was measured by densitometry. When the protein signal was so weak or undetectable, the background signal was used as a numeric quantification. The same square size was used to measure the loading control protein in each experiment. Each problem protein was normalized to loading control. Immunoblot images were representatives of different experiments. All graphs represent the ratio between the normalized protein and control condition.

### 4.8. Immunoprecipitation

Cells were incubated with 2 mM DSP (dithiobis(succinimidyl propionate), Thermo Fisher Scientific, Waltham, MA, USA) as a crosslinker for 30 min. The crosslinker reaction was stopped with 20 mM Tris pH 7.5 for 15 min. Cells were lysed with RIPA buffer and protease-phosphatase inhibitors. Proteins were collected by centrifugation for 15 min at 19,000× g at 4 °C and pre-incubated with Pierce™ Protein A/G Agarose (Thermo Fisher Scientific) beads for 1 h at 4 °C on a rotator and incubated with anti-FLAG^®^ or normal IgG antibodies for 2 h at 4 °C on a rotator. Antibody-bound proteins were incubated with beads overnight at 4 °C on a rotator. Beads were recovered by centrifugation at 1100× g and washed with cold RIPA buffer. Immunoprecipitated proteins were resolved by immunoblot using Clean-Blot™ IP Detection Kit HRP (Thermo Fisher Scientific). Antibodies are described in Appendix C.

### 4.9. Immunofluorescence and Confocal Fluorescence Microscopy

Cells were seeded on ᴓ 12 mm cover glasses (Thermo Fisher Scientific) in 24-well culture plates. After density conditions, cells were washed in PBS, fixed in 4% PFA (paraformaldehyde)-methanol-free (15710, Electron Microscopy Science, Hatfield, PA, USA) for 20 min and washed twice in PBS. Fixed cells were permeabilized with 0.3% Triton X100 in PBS (PBST) for 20 min. Cells were blocked with 1% BSA and 0.1% PBST for 1 h at RT. After blocking, cells were incubated with anti-YAP1 primary antibody in humidified chamber at RT for 2 h. Cells were washed three times in PBS and incubated with anti-rabbit IgG/Alexa-488 secondary antibody and f-actine staining (Alexa Fluor™ 647 Phalloidin, A22287, Thermo Fisher Scientific) for 1 h in a dark chamber at RT. Covers were washed in PBS and stained with 1 µg/mL DAPI (4′,6-diamidino-2-pheny-lindole, 268298, Merk Millipore, Burlington, MA, USA). Finally, cells were mounted with ProLong™ Glass Antifade Mountant (P36984, Thermo Fisher Scientific). Cells were visualized with a confocal microscopy system LSM710 with AxioObserver camera (Zeiss, Oberkochen, Germany). Antibodies are described in Appendix C.

### 4.10. RNA Isolation and qPCR Analysis

RNA was isolated from cells after lysis by TRIzol (Invitrogen), according to the manufacturer’s instructions. Total RNA was reverse transcribed using GoScript™ Reverse Transcriptase (Promega, Madison, WI, USA). Resulting cDNA was used for quantitative PCR amplification (qPCR) with specific primers by GoTaq^®^ qPCR Master Mix (Promega), according to the manufacturer`s instructions. Reaction was done by duplicate and values were normalized to housekeeping genes (*HPRT* or *GAPDH*). Relative mRNA levels were calculated in accordance to the point where each curve crossed the threshold cycle (Ct) from LyghtCycler v3.5 software (Roche) or ABI PRISM 7900HT SDS v2.4 software (Applied Biosystems) using the relative quantification (RQ) formula:

2^−ΔΔCt^ (ΔCt = Ct_gene_ − Ct_housekeeping_ and ΔΔCt = ΔCt_problem_ − ΔCt_control_)

Primers are described in Appendix D.

### 4.11. Drosophila Studies

Human DUSP10 gene was expressed in *Drosophila* imaginal discs using Gal4/UAS system [35]. DUSP10 cDNA was cloned into pUAS-WM vector with DUSP10 guides using Gateway™ Cloning Technology (Invitrogen), according to the manufacturer’s instructions. Transgenic UAS-hDUSP10-Myc (*nub-Gal4/+;UAS-DUSP10-Myc/+*) flies were done by embryonic microinjection [36]. sal^EPv^-Gal4 line was used to express human DUSP10 (sal^EPv^-Gal4/+;UAS-DUSP10-Myc/+) in the central domain of the wing blade [37]. DUSP10 gene expression was analyzed in flies with both UAS-hDUSP10-Myc and Gal4 constructs after conventional genetic crosses. UAS-Merlin-RNAi line and UAS-hppo^CA65a^ line was obtained from NIG-FLY (14228-R2, Mishima, Japan). For Myc epitope immunodetection in *Drosophila* wing discs and salivary gland cells, third instar larvae were fixed with 4% formaldehyde in PBST (0.1% Triton X100 in PBS) and incubated overnight at 4 °C with c-Myc antibody. Discs were incubated with a secondary antibody. Nuclei were stained with To-Pro-3 (T-3605, Thermo Fisher Scientific). Slides were prepared in Vectashield^®^ Mounting Medium (Vector Laboratories). Confocal images were captured by LSM510 confocal microscope from Optical and Confocal Microscopy Service of CBMSO. All images were processed using ImageJ software (NIH). Antibodies are described in Appendix C.

After expression of DUSP10-Myc during larval and pupal development at 29 °C, adult *Drosophila* females were selected for wing size measurements. At least 10 wings per group were mounted in ethanol:lactic acid (5:6 solution) for microscopic examination. Size of a wing region between the L5 vein and anterior wing margin was measured using Adobe Photoshop CS4.

### 4.12. Mouse Studies

Swiss nude (Crl:NU(Ico)-Foxn1^nu^) mice were purchased from Charles River Laboratory (Wilmington, MA, USA). HT29lucD6 derived cell line was injected subcutaneously (1.5 × 10^6^ cells/mouse) in female immunodeficient animals (7 weeks old) using 4–6 mice per group. Tumor growth was studied every week for 5–8 weeks by tumor volume and bioluminescence (BLI). Tumor volume was measured by a handheld caliper and estimated by ((width) ^2^ × (length))/2. BLI was measured at maximal luminescence emission after (D)-luciferin (Promega) injection (150 µg/gr mouse). BLI was acquired by IVIS Lumina II System (Caliper Life Sciences, Waltham, MA, USA) and quantified by Living Image^®^ in vivo imaging software (PerkinElmer, Waltham, MA, USA). Data results were normalized to first day post-inoculation for each animal and the mean of the group was presented.

### 4.13. Human Studies

Two different cohorts of tumor patient samples were collected from 73 and 999 paraffin-embedded tissue blocks from the Pathology Department of Amsterdam Medical Center (Amsterdam, The Netherlands) and the Surgery Department of Leiden University Medical Center (Leiden, The Netherlands), respectively. Sex, age, grade, status, Duke’s stage, and origin for each patient’s data were noted. A number of 32 and 38 samples in the first and second cohort, respectively, could not be analyzed by IHC from antibodies because of staining artefact, material loss, or few cells were detected.

TMA was dried, paraffin removed in xylene and rehydrated in ethanol (subsequently 100%, 90%, 70%, and 50%) washes. Endogenous peroxidase activity was blocked for 20 min with 0.3% H_2_O_2_-methanol. Antigen retrieval (0.01 M sodium citrate pH 6 with 0.1 M citric acid solution) was performed by boiling in a microwave for 10 min. Slides were incubated with primary antibodies diluted in 1% BSA in PBS at RT in a humidified chamber overnight. For each primary antibody, slides were stained simultaneously to avoid inter-experimental variations. Slides were incubated with biotinylated antibodies for 30 min at RT, using ABC Vectostain Standard Kit (Vectorlabs, Burlingame, CA, USA) and DAB solution (DAKO), according to the manufacturer’s instructions. Tissue was contrastained with hematoxylin (Millipore) and mounted with Entellan New (Millipore). Two independent observers (M.J.-M. and L.R.v.d.B.) used criteria proposed [38] in order to quantify COX2, YAP1, and DUSP10 staining. For phospho-p38 scoring, tissue samples were categorized by positive or negative labeling. Antibodies are described in Appendix C.

### 4.14. Study Approval

The study protocol for the 79 and 999 patient cohorts was collected and supervised by the Pathology Department of Amsterdam Medical Center (Amsterdam, The Netherlands) and the Surgery Department of Leiden University Medical Center (Leiden, The Netherlands), respectively. 

All animal experimentation studies complied with the National and European Union Legislation and was supervised by the Ethics Committee of CBMSO (Madrid, Spain).

### 4.15. Statistics

Student’s *t*-test or two-way ANOVA followed by Bonferroni’s post-test were used depending on the experiment. Statistical analyses were performed using GraphPad Prism5 software (https://www.graphpad.com/scientific-software/prism/).

Statistical analysis of the TMA was performed using SPSS software version 23 (IBM). To compare categorical data, we used two-way ANOVA followed by Fisher’s exact correction. The relationship between nuclear DUSP10 staining and tumor stage was analyzed using an ordinal logistic regression model, with nuclear DUSP10 intensity as the independent and tumor stage as the dependent variable, adjusting for age (continuous), gender (man/woman), and chemotherapy (yes/no). Cox proportional hazard models were used to study the association of nuclear DUSP10 staining with overall survival, with adjustment for age, gender, chemotherapy, and tumor stage as potential confounding factors. Statistical analysis of the *Drosophila* model was performed using Student’s *t*-test on Microsoft Excel software. Results were considered statistically significant when *p*-value was <0.05 (* *p* < 0.05; ** *p* < 0.01; *** *p* < 0.001).

## 5. Conclusions

Our findings describe a novel role of DUSP10 on the regulation of YAP1 and its cellular localization, avoiding CCI. DUSP10 is required for in vitro proliferation, in vivo tumor xenograft growth in CRC lines, and rescuing the altered *hippo* signaling in *Drosophila*. We propose DUSP10 avoids CCI through YAP1 in stress conditions such as HD. DUSP10 activity on YAP1 may be dual: (1) DUSP10 would bind Ser397 YAP1 and promote its dephosphorylation in the cytoplasm, avoiding its degradation; and (2) DUSP10, independently of its phosphatase activity, would also favor nuclear YAP1 retention. The importance of this mechanism is strongly supported by nuclear DUSP10 and nuclear YAP1 presence in tumor tissue of colon cancer patients; and more importantly, poor prognosis is related to high nuclear DUSP10 expression.

## Figures and Tables

**Table 1:** Analysis of tumor epithelial tissue from colon cancer patients according to protein expression. COX2, DUSP10, YAP1, and p-p38 expression are analyzed in tumor epithelial tissue from a cohort of 73 colon cancer patients. Two-way ANOVA analysis was performed.

			DUSP10 Cytoplasmic (*n*= 67)			DUSP10 Nuclei (*n*= 67)		
Characteristic		No. of Patients ^a^	Weak	Strong	*p*-Value ^b^	Weak	Strong	*p*-Value ^b^
**COX2 cytoplasmic, No. (%)**								
	Weak	22 (32.8)	8 (61.5)	14 (25.9)	**0.022**	-	-	-
	Strong	45 (67.2)	5 (38.5)	40 (74.1)		-	-	-
**YAP1nuclei, No. (%)**								
	Weak	18 (28.6)	-	-	-	8 (61.5)	10 (20.0)	**0.006**
	Strong	45 (71.4)	-	-	-	5 (38.5)	40 (80.0)	
**p-p38 staining, No. (%)**								
	Positive	2 (3.1)	0 (0.0)	2 (3.7)	1.0	0 (0.0)	2 (3.9)	1.0
	Negative	62 (96.9)	10 (100.0)	52 (96.3)		13 (100.0)	49 (96.1)	

^a^ 10, 9 and 6 of 73 samples could not be analyzed for COX2, YAP1, p-p38 and DUSP10, respectively. ^b^ Boldface type indicates statistical significance (*p* < 0.05).

**Table 2:** Analysis of DUSP10 expression in colon cancer tissue according to clinical-pathological categories. DUSP10 expression was analyzed in tumor epithelial tissue from a cohort of 999 patients depending on clinical-pathological categories (sex, Dukes’ stage, grade of differentiation, origin, or patient’s status). Two-way ANOVA analysis was performed to study the patient variation.

			Cytoplasmic DUSP10 (*n* = 961 )			Nuclear DUSP10 (*n* = 961)		
Categories		No. of Patients ^a^	Weak	Strong	*p*-Value ^b^	Weak	Strong	*p*-Value ^b^
**Sex, No. (%)**								
	Female	479 (49.8)	293 (51.7)	186 (47.2)	0.190	235 (47.7)	244 (52.1)	0.176
	Male	482 (50.2)	274 (48.3)	208 (52.8)		258 (52.3)	224 (47.9)	
**Age, No. (%)**								
	≤65	329 (34.2)	199 (35.1)	130 (33.0)	0.457	155 (31.4)	174 (37.2)	0.156
	66-74	292 (30.4)	172 (30.3)	120 (30.5)		159 (32.3)	133 (28.4)	
	≥75	340 (35.4)	196 (34.6)	144 (36.5)		179 (36.3)	161 (34.4)	
**Stage, No. (%)**								
	I	136 (14.2)	88 (15.6)	48 (12.2)	0.391	80 (16.2)	56 (12.0)	**0.043**
	II	392 (40.9)	232 (41.1)	160 (40.7)		201 (40.8)	191 (41.1)	
	III	271 (28.3)	147 (26.0)	124 (31.6)		140 (28.4)	131 (28.2)	
	IV	159 (16.6)	98 (17.3)	61 (16.6)		72 (14.6)	87 (18.7)	
	Unknown	3 (0.3)						
**Grade, No. (%)**								
	Well differentiated	96 (10.0)	50 (8.8)	46 (11.7)	**0.027**	53 (10.8)	43 (9.2)	0.185
	Moderately differentiated	607 (63.2)	348 (61.4)	259 (65.7)		293 (59.4)	314 (67.1)	
	Poorly differentiated	182 (18.9)	118 (20.8)	64 (16.2)		108 (21.9)	74 (15.8)	
	Unknown	76 (7.9)						
**Chemotherapy, No. (%)**								
	No	673 (70.0)	412 (72.7)	261 (66.2)	**0.038**	352 (71.4)	321 (68.6)	0.360
	Yes	288 (30.0)	155 (27.3)	133 (33.8)		141 (28.6)	147 (31.4)	
**Radiotherapy, No. (%)**								
	No	948 (98.6)	557 (98.2)	391 (99.2)	0.259	485 (98.4)	463 (98.9)	0.580
	Yes	13 (1.4)	10 (1.8)	3 (0.8)		8 (1.6)	5 (1.1)	

^a^ 38 samples could not be analyzed for DUSP10. ^b^ Boldface type indicates statistical significance (*p* < 0.05).

**Table 3:** Survival analysis of colon cancer patients depending on nuclear DUSP10 protein expression. A cohort of 999 colon cancer patients was analyzed and classified by weak and strong staining for DUSP10. Number of events (deaths per group) were quantified. The table represents the relative hazard ratio (RR) and 95% confidence interval (CI).

Nuclear DUSP10		No. of Patients	No. of Death	RR Univariate (95% CI)	*p*-Value ^a^	RR Adjusted ^1^ (95% CI)	*p*-Value ^a^	RR Adjusted ^2^ (95% CI)	*p*-Value ^a^
Weak		481	211	1 [Reference]	**0.047**	1 [Reference]	**0.033**	1 [Reference]	**0.189**
Strong		459	213	1.22 (1.00–1.49)		1.24 (1.02–1.51)		1.14 (0.94–1.39)	

^a^ Boldface type indicates statistical significance (*p* < 0.05). ^1^ Cox multivariate analysis adjusted to sex and chemotherapy confounders (RR1). ^2^ Cox multivariate analysis adjusted to sex, chemotherapy and tumor stage cofounders (RR2).

## Supplementary Materials

The following are available online at https://www.mdpi.com/2072-6694/11/11/1767/s1, Figure S1: DUSP10 expression regulates p38 dephosphorylation in HT29lucD6 cell line, Figure S2: DUSP10 overexpression in HCT116 cell line promotes in vitro and in vivo growth, Figure S3: High density effects in HCT116 cell line, Figure S4: Immunofluorescence staining for YAP1 protein at low and high density in CRC cell lines, Figure S5: A YAP inhibitor in preventing DUSP10-enhanced proliferation in CRC cell line, Figure S6: Transient transfection of YAP and DUSP10 mutant plasmids in HCT116, **Figure S7**: In silico analysis of DUSP10 expression in CRC patients, Figure S8: Immunoblots related to Figure 2b,d, Figure S9: Immunoblots related to Figure 2e,f, Figure S10: Immunoblot related to Figure 3b, Figure S11: Immunoblot related to Figure 4a,b, Figure S12: Immunoblot related to Figure 4c.

## Appendix A

Plasmids:

pcDNA-FLAG was obtained from our laboratory’s repository.

pcDNA-YAP1-FLAG (Addgene plasmid #18881) was a gift from Yosef Shaul [39].

pCMV-YAP S381A-FLAG (Addgene plasmid #27377) were a gift from Kunliang Guan [9].

pCMV-YAP S127A-FLAG (Addgene plasmid #27370) were a gift from Kunliang Guan [18].

pLenti-CMV/TO-Hygro empty (w214-1) (Addgene plasmid #17484) was a gift from Eric Campeau and Paul Kaufman [40].

pLenti-KO.1-Puro carrying shRNA DUSP10 sequences (TRCN0000001885/1886/1889) from Sigma.

pLX304-V5 plasmid (Addgene plasmid #25890) was a gift from David Root [41].

8xGTII/Luc reporter (Addgene plasmid # 34615) was a gift from Stefano Piccolo [42].

SV40-Renilla was obtained from our laboratory’s repository.

YAP esiRNA Mission (Sigma #EHU113021) and Universal Negative Control siRNA Mission (Sigma #SIC001).

## Appendix B

Guides for cloning:

*DUSP10* (in pLenti-Hygro vector): sense ^5′^gtggatccatgcctccgtctcctttag^3′^ and anti-sense ^5′^cactctagattcacacaaccgtctccac^3′^ from Sigma.

*DUSP10* (in pLX304-V5 and pUAS-WM): sense ^5′^caccatgcctccgtctcctttagacgac^3′^ and anti-sense ^5′^cacaaccgtctccacgcccatcag^3′^ from Sigma.

*DUSP10*-C408S mutation: sense ^5′^gggcttctcatccactcccaggctggggtgtcc^3′^ and anti-sense ^5′^ggacaccccagcctgggagtggatgagaagccc^3′^ from Sigma.

DUSP10-AA mutation: sense ^5′^gggcttctcatccactcccaggctggggtgtcc^3′^ and anti-sense ^5′^ggacaccccagcctgggagtggatgagaagccc^3′^ from Sigma.

## Appendix C

Antibodies:

DUSP10 #3483 from Cell Signaling Technology. (*Western Blot*)

DUSP10 ab140123 from Abcam. (*Immunohistochemistry*)

phospho-p38 (3D7) #9215 from Cell Signaling Technology. (*Western Blot and Immunohistochemistry*)

p38 #9212 from Cell Signaling Technology. (*Western Blot*)

V5-Tag (D3H8Q) #13202 from Cell Signaling Technology. (*Western Blot*)

YAP1 (D8H1X) #14074 from Cell Signaling Technology. (Western Blot, Immunofluorescence and Immunohistochemistry)

phospho-YAP Ser397 (D1E7Y) #13619 from Cell Signaling Technology. (*Western Blot*)

phospho-YAP Ser127 (D9W21) #13008 from Cell Signaling Technology. (*Western Blot*)

HSP90α/β (H-114) #sc7947 from Santa Cruz Biotechnology. (*Western Blot*)

phospho-JNK (G-7) #sc6254 from Santa Cruz Biotechnology. (*Western Blot*)

JNK1/2/3 (FL) #sc474 from Santa Cruz Biotechnology. (*Western Blot*)

C-Myc (9E10): sc-40 from Santa Cruz Biotechnology. (*Immunofluorescence*)

GAPDH #MAB374 from Millipore. (*Western Blot*)

LAMIN A/C (131C3) #ab8984 from Abcam. (*Western Blot*)

FLAG^®^ M2 #F1804 from Sigma. (Western Blot and Immunoprecipitation)

α-TUBULIN (DM1A) #T6199 from Sigma. (*Western Blot*)

Normal mouse IgG #12-371 from EMD Millipore. (*Immunoprecipitation*)

Mouse HRP secondary antibody: #7076 from Cell Signaling. (*Western Blot*)

Rabbit HRP secondary antibody: #7074 from Cell Signaling. (*Western Blot*)

Rabbit Biotinylated secondary antibody: E0432 from DAKO. (*Immunohistochemistry*)

Mouse Biotinylated secondary antibody: E0433 from DAKO. (*Immunohistochemistry*)

Alexa-conjugated secondary antibody: A-31570 from Invitrogen. (*Immunofluorescence*)

## Appendix D

Primers for quantitative PCR:

*HPRT*: sense ^5′^ctggaaagaatgtcttgattgtgg^3′^ and anti-sense ^5′^catctttggattatactgcctgac^3′^ from Sigma.

*GAPDH:* sense ^5′^gcacaagaggaagagagagagacc^3′^ and anti-sense ^5′^aggggagattcagtgtggtg^3′^ from Sigma.

*DUSP10: sense*^5′^cccagccacttcacatagtc^3′^ and anti-sense ^5′^tactaagtccacctttcaacacc^3′^ from Sigma.

*YAP1:* sense ^5′^aatcccactcccgacagg^3′^ and anti-sense ^5′^gactactccagtgggggtca^3′^ from Sigma.
