# Supplementary material for: DUSP10 Is a Regulator of YAP1 Activity Promoting Cell Proliferation and Colorectal Cancer Progression
Source: Cancers (Basel). 2019 Nov 9;11(11):1767. doi: 10.3390/cancers11111767 (PMC6896144; doi:10.3390/cancers11111767)
Supplement: Supplementary file 1 [file cancers-11-01767-s001.pdf]

Supplementary Material

DUSP10 is a Regulator of YAP1 Activity Promoting Cell Proliferation and Colorectal Cancer Progression

Marta Jiménez-Martínez, Cristina M. Ostalé, Lennart R. van der Burg, Javier Galán-Martínez, James C.H. Hardwick, Ricardo López-Pérez, Lukas J.A.C. Hawinkels, Konstantinos Stamatakis and Manuel Fresno

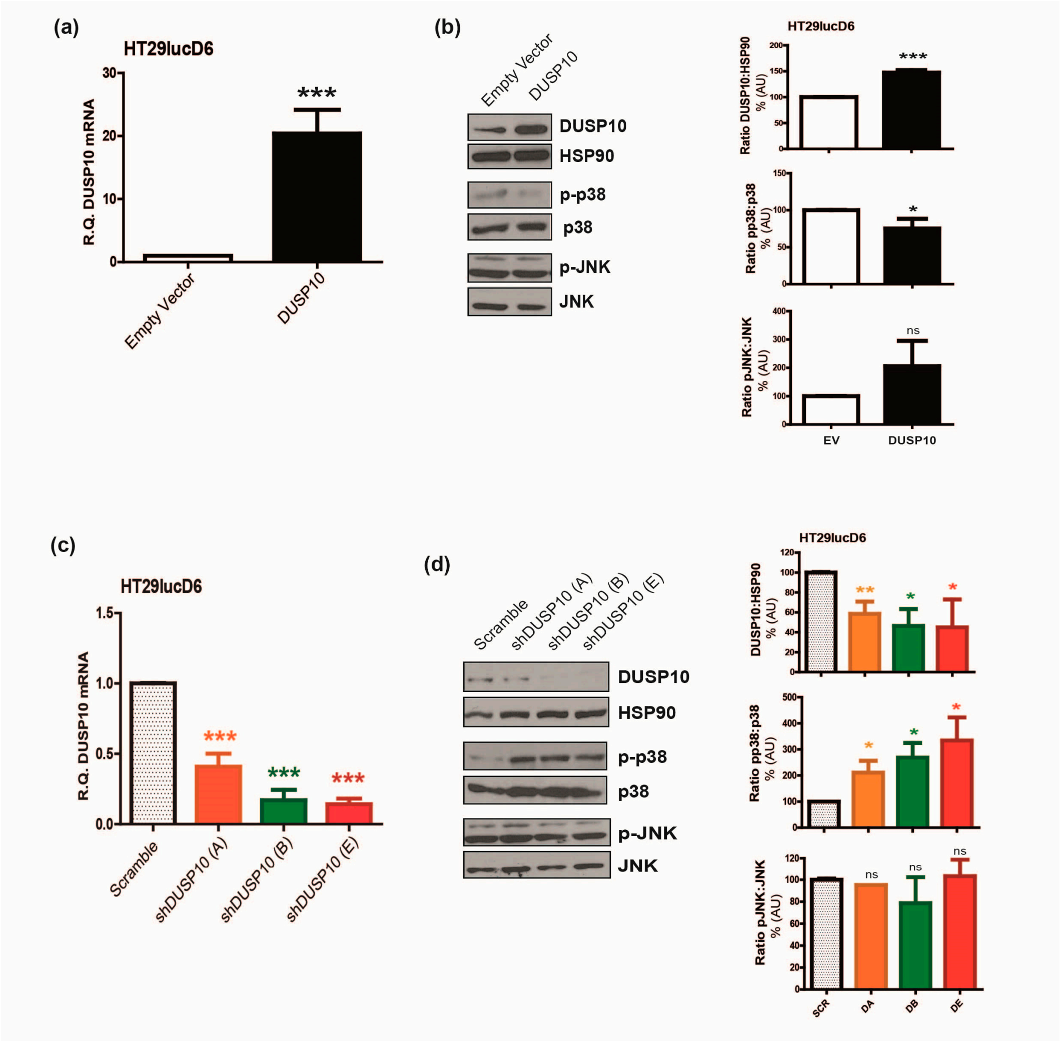

Figure S1. DUSP10 expression regulates p38 dephosphorylation in HT29lucD6 cell line. (**a**) *DUSP10* mRNA expression in HT29lucD6-DUSP10 compared to HT29lucD6-EV. Student's t-test (mean ± SEM; ***$p < 0.001$) and 9 independent experiments were performed. (**b**) Expression of DUSP10, p-p38, p38, p-JNK and JNK protein levels in HT29lucD6-DUSP10 and HT29lucD6-EV. *(Left)* A representative image of 3 independent experiments. *(Right)* Quantification of those all blots performed (mean ± SEM; Student's t-test; *$p < 0.05$, ***$p < 0.001$, *ns= no significance*). (**c**) *DUSP10* mRNA expression in HT29lucD6-shDUSP10 compared to HT29lucD6-SCR. Student's t-test (mean ± SEM; ***$p < 0.001$) and 6 independent experiments were performed. (**d**) Expression of DUSP10, p-p38, p38, p-JNK and JNK protein levels in HT29lucD6-DUSP10 and HT29lucD6-SCR. *(Left)* A representative image of 3 independent experiments. *(Right)* Quantification of those all blots performed (mean ± SEM; Student's t-test; *$p < 0.05$, **$p < 0.01$, *ns= no significance*).

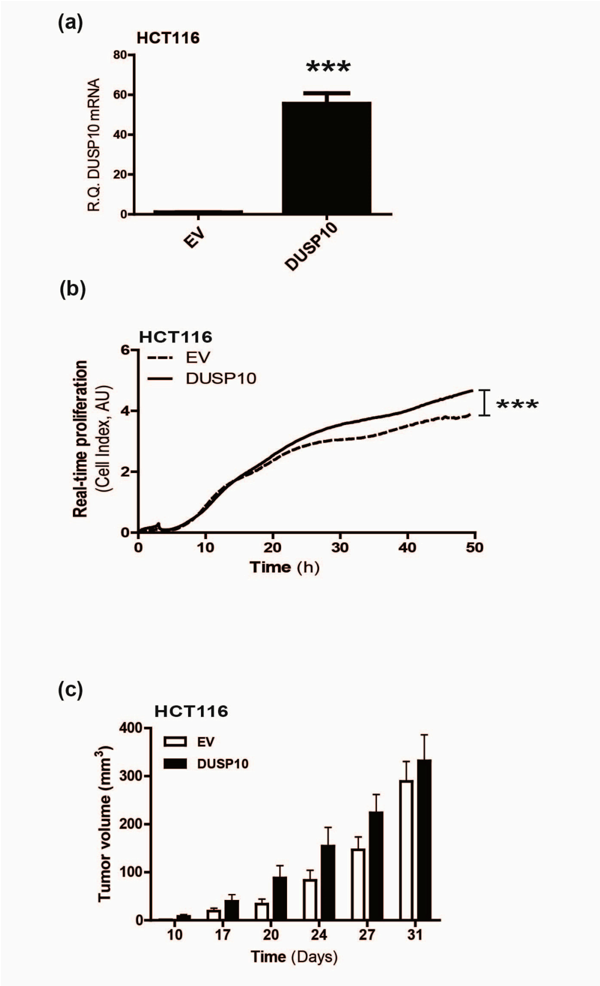

Figure S2. DUSP10 overexpression in HCT116 cell line promotes in vitro and in vivo growth. (**a**) Analysis of *DUSP10* mRNA expression in HCT116-DUSP10 and HCT116-EV at exponential proliferation stage. Student's t-test (media ± SEM; ***$p < 0.001$) and 3 independent experiments were performed. (**b**) Growth curves of HCT116-EV and HCT116-DUSP10 for 50 hours using real-time proliferation analysis by xCELLigence technology. Linear regression analysis was performed ($p < 0.05$). Representative graph of 2 independent experiments. (**c**) Tumor volume of HCT116-DUSP10 and HCT116-EV xenografts was measured for 4 weeks. Two-way ANOVA followed by Bonferroni's multiple comparison tests were performed (mean ± SEM; $p < 0.05$; 6-7 mice per group).

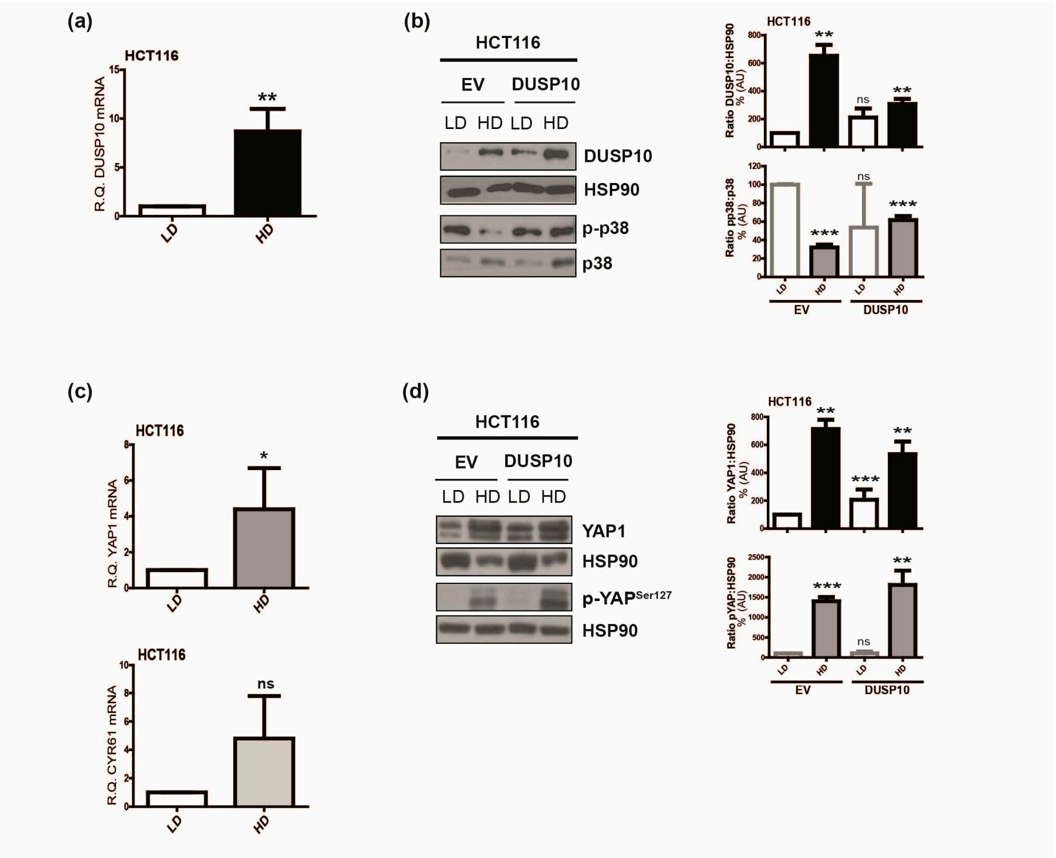

Figure S3. High density effects in HCT116 cell line. (**a**) *DUSP10* mRNA was quantified of HCT116 in low-density (LD) and high-density (HD). Student's t-test (mean ± SEM; ***p < 0.01*) and 4 independent experiments were performed. (**b**) Expression of DUSP10 and p-p38 of HCT116 in LD and HD. (Left) A representative image of 3 independent experiments. *(Right)* Quantification of those all blots performed (mean ± SEM; Student's t-test; ***p < 0.01, ***p < 0.001, ns = no significance*). (**c**) *YAP1* and *CYR61* mRNA were quantified of HCT116 in LD and HD. Student's t-test (mean ± SEM; **p < 0.05, ns = no significance*) and 5 independent experiments were performed. (**d**) Expression of YAP1 and p-YAPSer127 of HCT116 in LD and HD. (Left) A representative image of 3 independent experiments. (Right) Quantification of those all blots performed (mean ± SEM; Student's t-test; ***p < 0.01, ***p < 0.001, ns = no significance*).

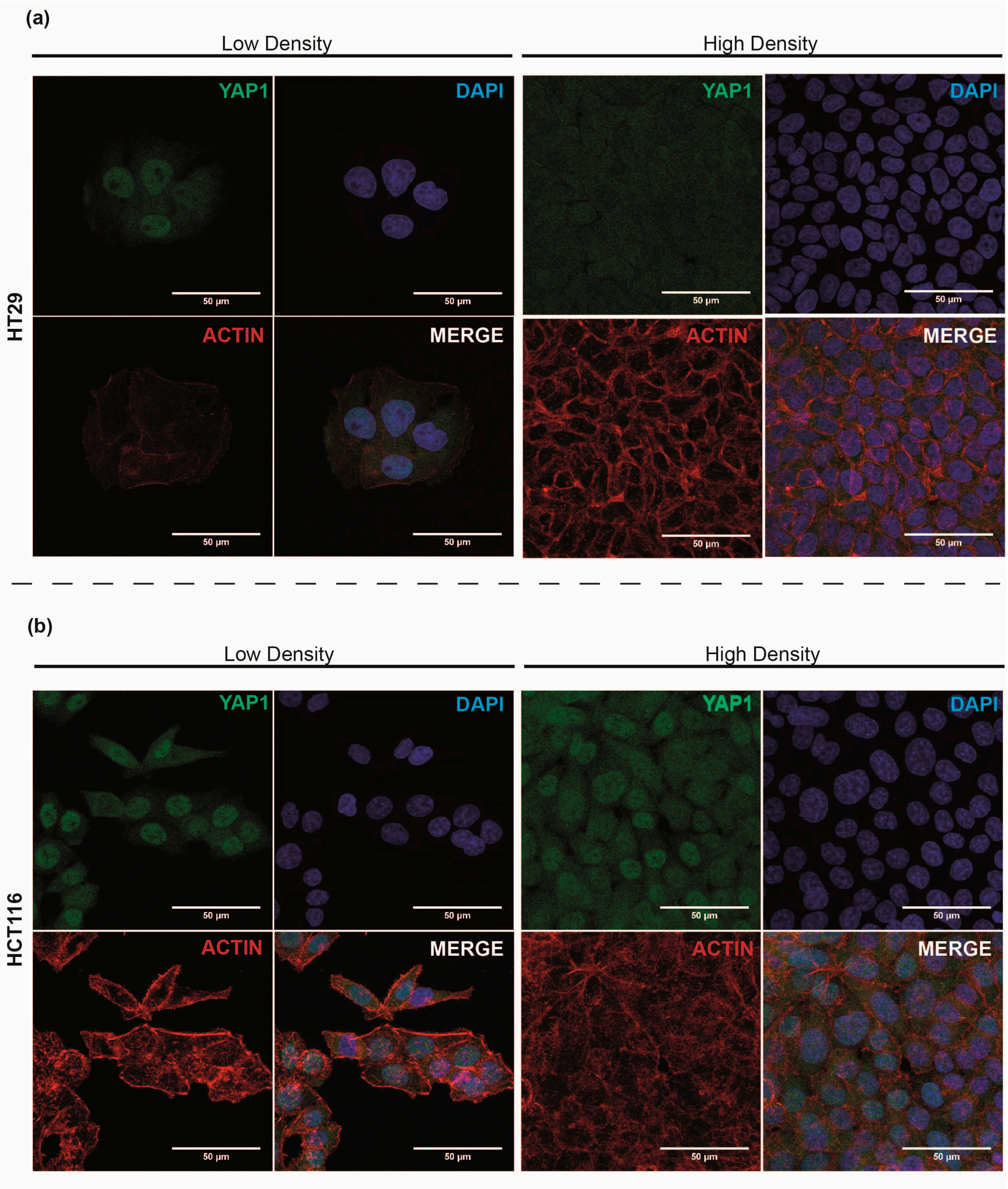

Figure S4. Immunofluorescence staining for YAP1 protein at low and high density in CRC cell lines. YAP1 protein (green), nuclei staining (DAPI, blue) and f-actin (phalloidin, red) were detected by IF using a confocal microscope. These are a representative confocal images at LD and HD of HT29 (**a**) and HCT116 (**b**). Bars, 50 µm.

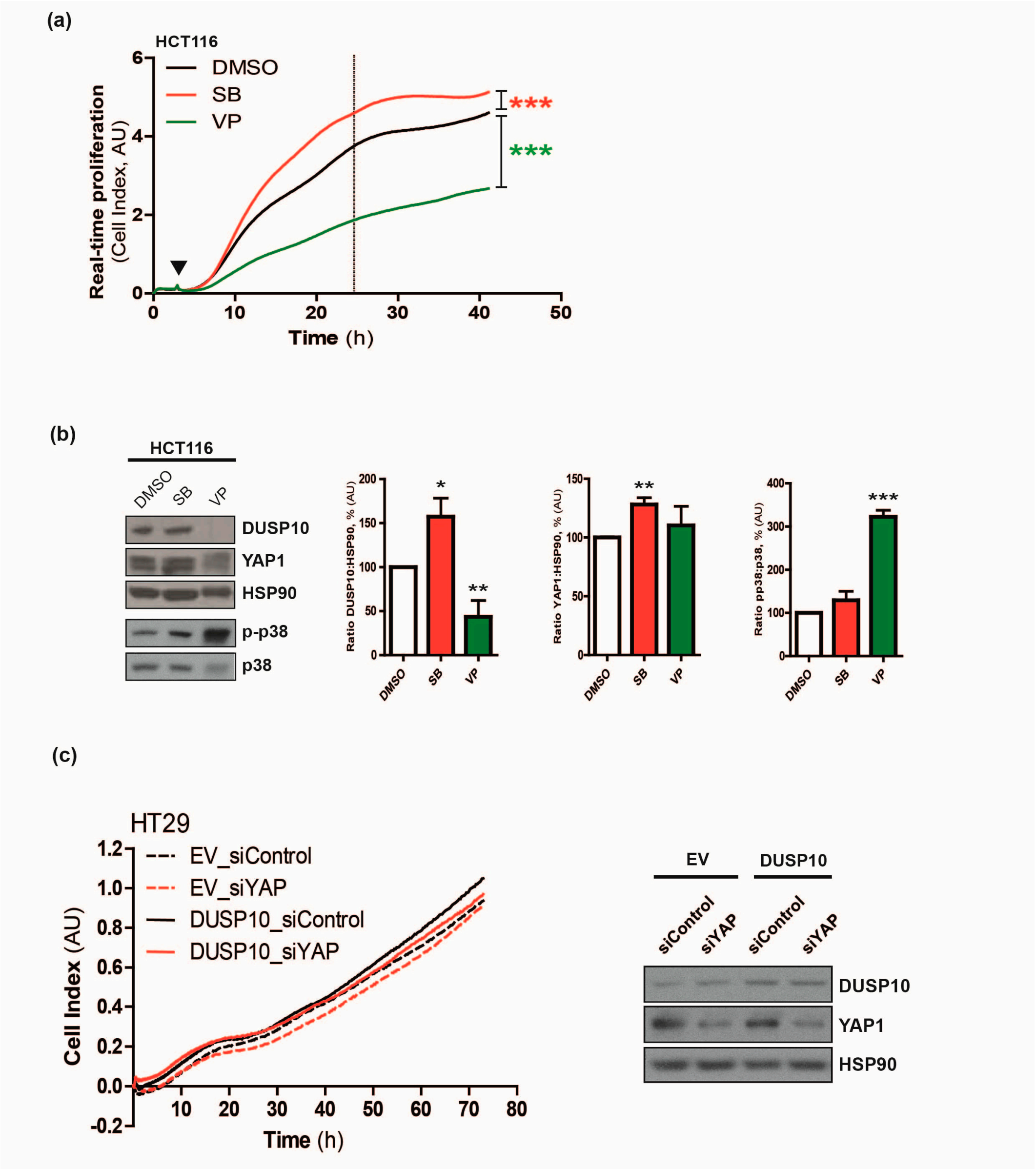

Figure S5. A YAP inhibitor in preventing DUSP10-enhanced proliferation in CRC cell lines. (**a**) Growth curves of HCT116 proliferative response treated with SB239063 (SB 1 µM) and verteporfin (VP 1 µM) for 40 hours after 2 hours seeding (▼) using real-time proliferation analysis by xCELLigence technology. Linear regression analysis was performed (***$p < 0.001$). Representative graph of 2 independent experiments. (**b**) Expression of DUSP10, YAP1, p-p38 and p38 protein levels in HCT116 treated with SB (1 µM) and VP (1 µM) for 24 hours (corresponding dotted line within graphic of Figure S4A). *(Left)* A representative image of 4 independent experiments. *(Right)* Quantification of those all blots performed (mean ± SEM; Student's t-test; *$p < 0.05$, **$p < 0.01$, ***$p < 0.001$). (**c**) Growth curves of HT29 proliferative response for 72h after EV and DUSP10 overexpressing cell line were transiently transfected with siRNA Negative Control or siRNA YAP using real-time proliferation analysis by xCELLigence technology. Linear regression analysis was performed ($p < 0.05$). (*Left*) Representative graph of 2 independent experiments. (*Right*) image represented DUSP10 and YAP1 expression levels of used cell lines at initial proliferation time.

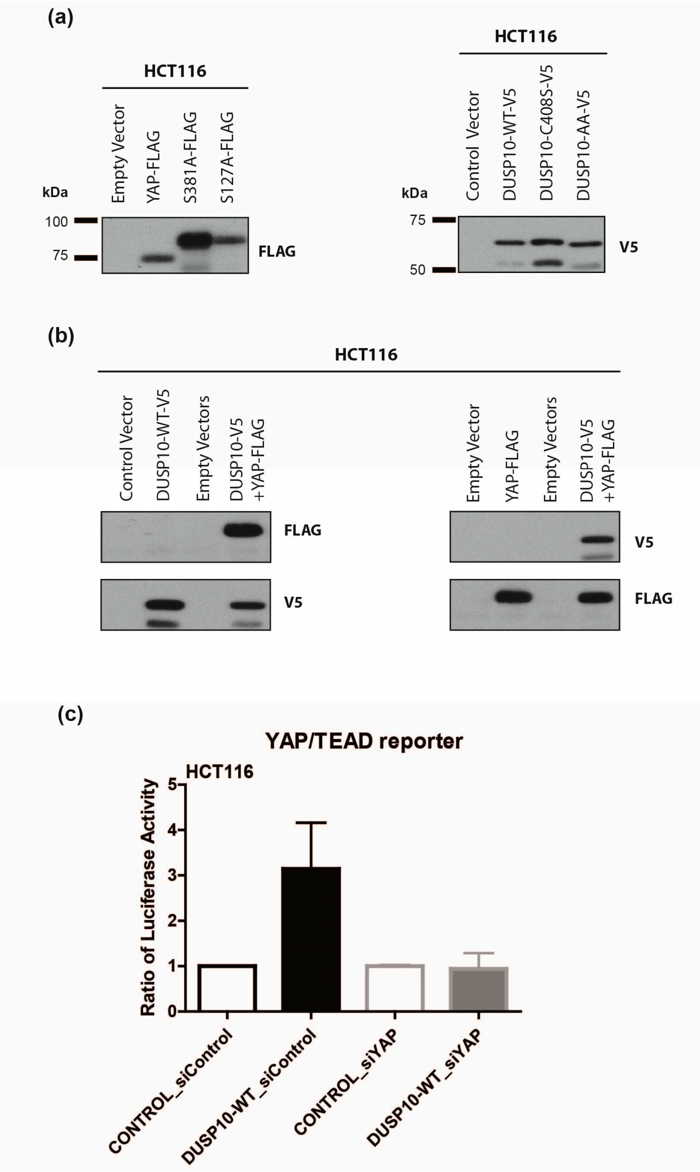

Figure S6. Transient transfection of YAP and DUSP10 mutant plasmids in HCT116. (**a**) Expression of YAP and DUSP10 mutants overexpressed into HCT116 cell line. (Left representative image) YAP (YAP-FLAG), S381A YAP mutant (S381A-FLAG) and S127A YAP mutant (S127A-FLAG) expression and its empty vector plasmid were detected with anti-FLAG antibody after to transfect for 48 hours. *(Right representative image)* DUSP10-WT, DUSP10-C408S and DUSP10-AA expression and its control plasmid were detected with anti-V5 antibody after to transfect for 48 hours. (**b**) Co-transfection of DUSP10-V5 and YAP-FLAG expression plasmids into HCT116 for 48 hours. Detection DUSP10 and YAP overexpression using anti-V5 and anti-FLAG antibodies, respectively. (**c**) Relative luciferase activity of the 8xGTII-luc (YAP/TEAD binding element reporter) was measured, responding to DUSP10 overexpression (DUSP10-WT) and siRNA YAP (siYAP). HCT116 was transiently transfected with the indicated plasmids and its control constructs.

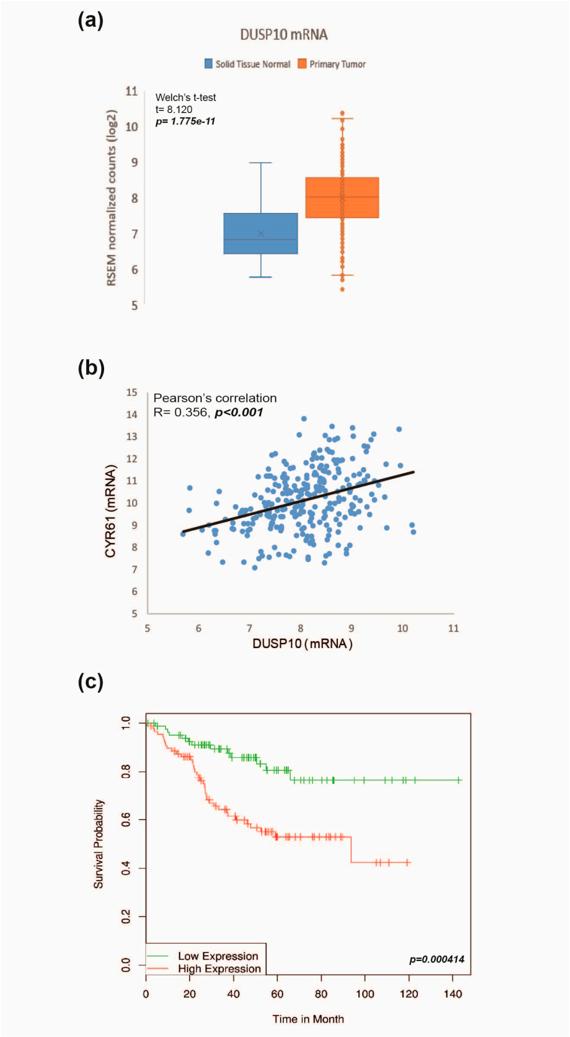

Figure S7. In silico analysis of DUSP10 expression in CRC patients. (**a**) *DUSP10* mRNA was analyzed in normal tissue (blue) and primary CRC tissue (orange) of 430 patients from TCGA of CRC cohort. Pearson's correlation was performed. (**b**) Correlation analysis of *DUSP10* with *CYR61* (YAP1 target gene) mRNA expression were performed of 430 patients from TCGA of CRC cohort. Pearson's correlation was performed. (**c**) Survival analysis related to *DUSP10* mRNA gene expression (low, *n* = 83; high, *n* = 84) profile in colon cancer metastasis patients (*n* = 167) with 225501_s_at reporter from GSE17538 database. One-way ANOVA comparison statistical test was performed.

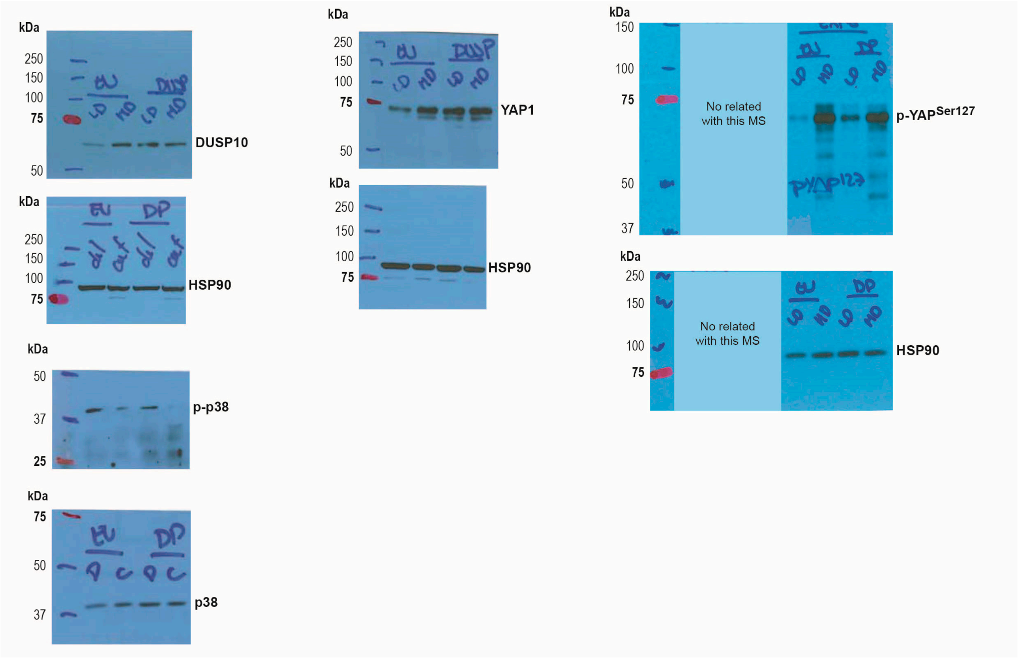

Figure S8. Immunoblots related to figure 2b and 2d.

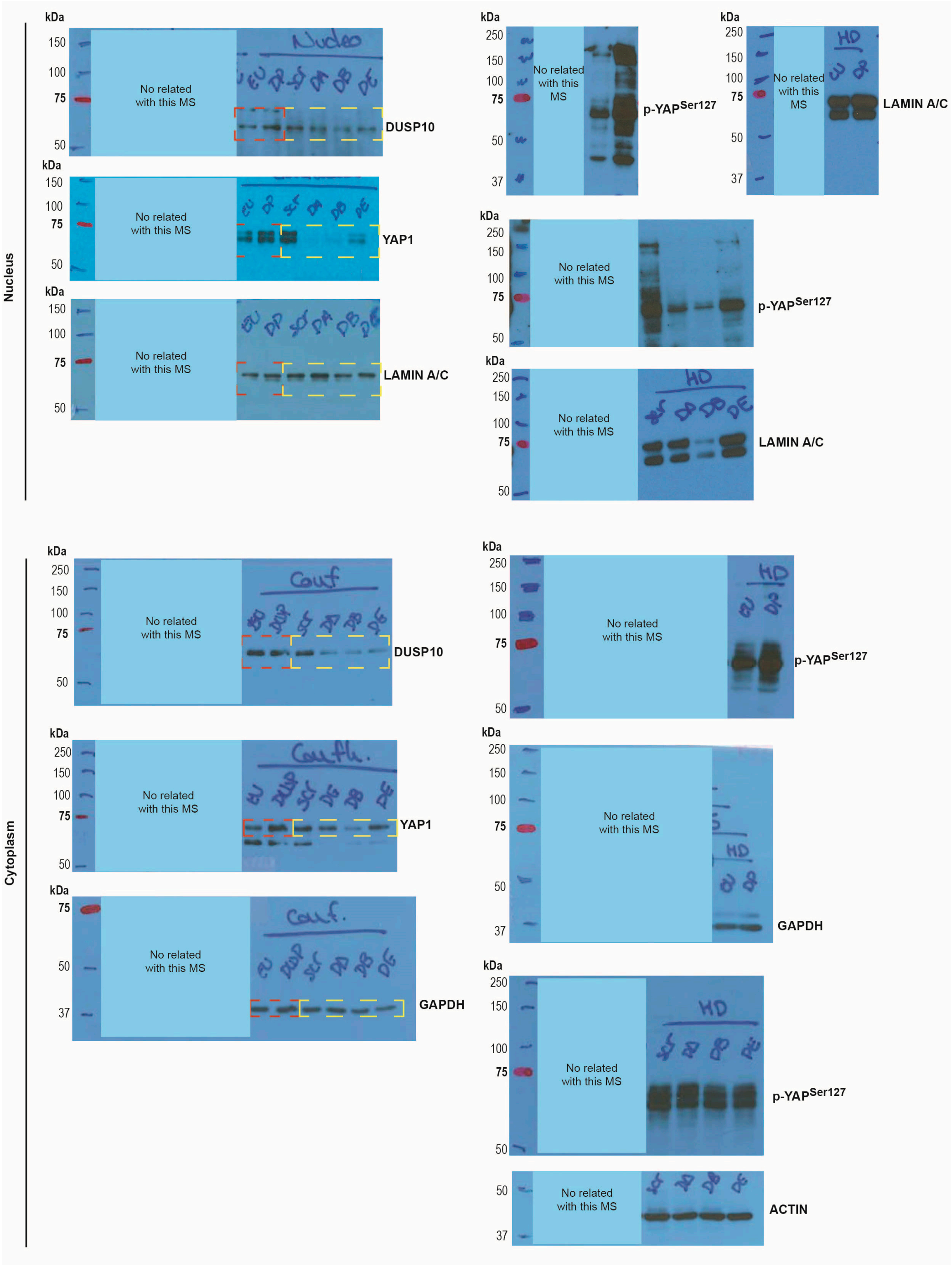

Figure S9. Immunoblots related to figure 2e and 2f.

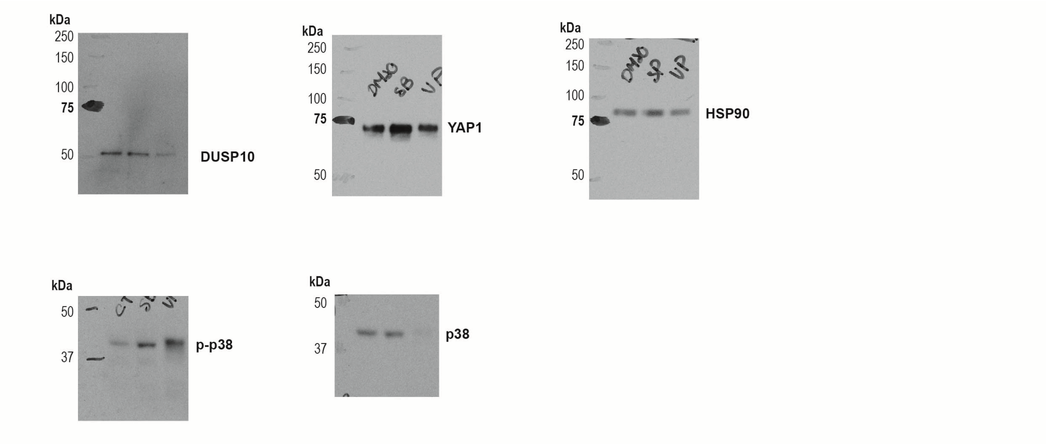

Figure S10. Immunoblots related to figure 3b.

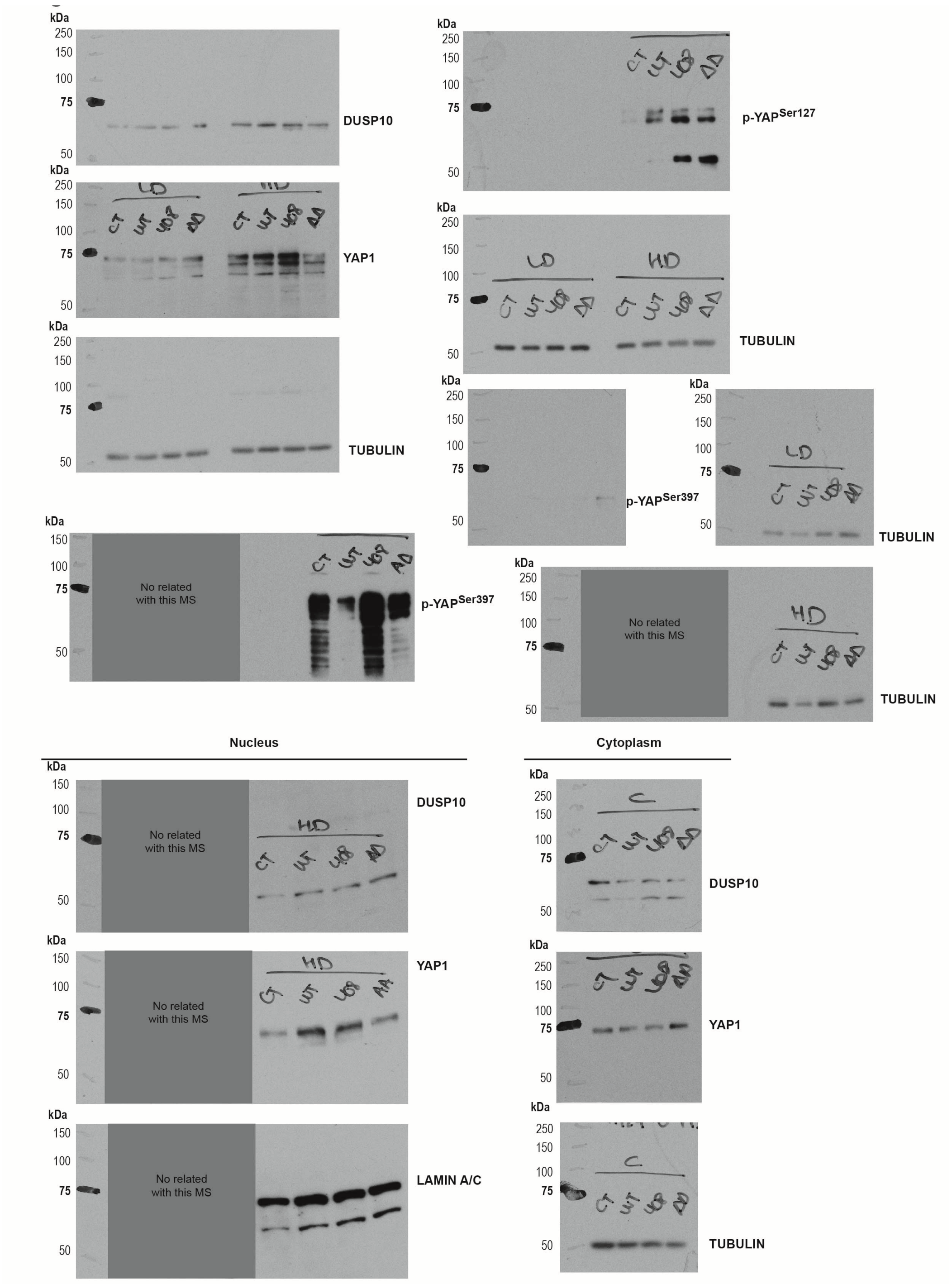

Figure S11. Immunoblots related to figure 4a and 4b.

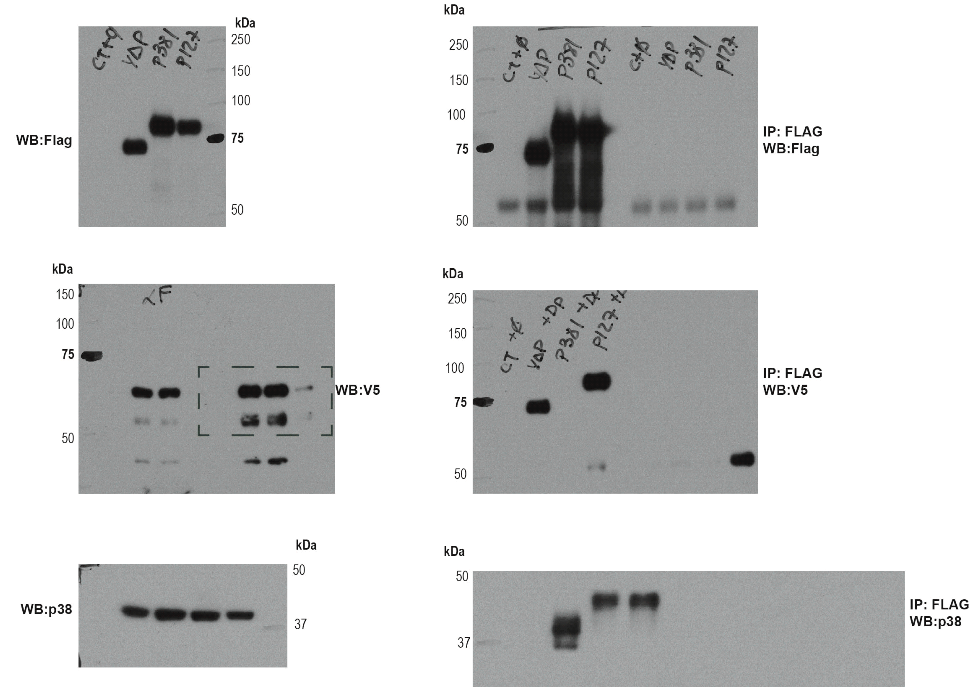

Figure S12. Immunoblots related to figure 4c.

© 2019 by the authors. Licensee MDPI, Basel, Switzerland. This article is an open access article distributed under the terms and conditions of the Creative Commons Attribution (CC BY) license (http://creativecommons.org/licenses/by/4.0/).
